# Differential analysis of combinatorial protein complexes with CompleXChange

**DOI:** 10.1186/s12859-019-2852-z

**Published:** 2019-06-03

**Authors:** Thorsten Will, Volkhard Helms

**Affiliations:** 1 Center for Bioinformatics, Saarland University, Campus E2.1, Saarbrücken, 66123 Germany; 2 Graduate School of Computer Science, Saarland University, Campus E1.3, Saarbrücken, 66123 Germany

**Keywords:** Differential protein complex analysis, Protein complex abundance estimation, Transcription factor complexes, Differential analysis, Monocytes

## Abstract

**Background:**

Although a considerable number of proteins operate as multiprotein complexes and not on their own, organism-wide studies so far are only able to quantify individual proteins or protein-coding genes in a condition-specific manner for a sizeable number of samples, but not their assemblies. Consequently, there exist large amounts of transcriptomic data and an increasing amount of data on proteome abundance, but quantitative knowledge on complexomes is missing. This deficiency impedes the applicability of the powerful tool of differential analysis in the realm of macromolecular complexes. Here, we present a pipeline for differential analysis of protein complexes based on predicted or manually assigned complexes and inferred complex abundances, which can be easily applied on a whole-genome scale.

**Results:**

We observed for simulated data that results obtained by our complex abundance estimation algorithm were in better agreement with the ground truth and physicochemically more reasonable compared to previous efforts that used linear programming while running in a fraction of the time. The practical usability of the method was assessed in the context of transcription factor complexes in human monocyte and lymphoblastoid samples. We demonstrated that our new method is robust against false-positive detection and reports deregulated complexomes that can only be partially explained by differential analysis of individual protein-coding genes. Furthermore we showed that deregulated complexes identified by the tool potentially harbor significant yet unused information content.

**Conclusions:**

CompleXChange allows to analyze deregulation of the protein complexome on a whole-genome scale by integrating a plethora of input data that is already available. A platform-independent Java binary, a user guide with example data and the source code are freely available at https://sourceforge.net/projects/complexchange/.

**Electronic supplementary material:**

The online version of this article (10.1186/s12859-019-2852-z) contains supplementary material, which is available to authorized users.

## Background

Cellular function is a team effort because proteins rarely perform their biochemical tasks all alone. Instead, proteins frequently collide with other gene products in the crowded environment of the cell, they may selectively bind to other proteins driven by physical interactions, they may dynamically assemble into complexes in a well-coordinated manner and accomplish their tasks cooperatively [1, 2]. Such multiprotein complexes may be either clearly defined modules of interaction partners that represent permanently assembled molecular machines or combinatorial formations of transient interaction partners in a dynamic interplay [3–5].

Whereas the experimental detection of protein complexes is generally speaking a mature field, it is still time-consuming and subject to high false-discovery rates. Quantitative profiling of the complete complexome in a condition-specific way is currently not feasible in a high-throughput fashion [6–9]. More so, direct quantitative measures are limited to a definite protein space and only cover pairwise complexation [10–13].

Nowadays a plethora of data on gene expression and an increasing amount of data on proteome abundances enable to also approach the dynamics of the condition-specific complexome by computational methods. Guided by static compilations of protein interactions, the correlation of gene expression or protein abundance between putative interaction partners was used as a proxy to study their collective behavior [4, 14, 15]. Besides, the topic was examined by integrating expression data with known protein complexes [3] and annotated pathways [16]. However, such simplified models lack a ruleset addressing how proteins that are expressed in low amounts and that are shared between different binding partners may limit complex formation. Approaches dealing with such interdependencies and the limitedness of gene products have been attempted by stochastic simulations with according computational effort [17] and by linear optimization on fixed sets of reference complexes [18, 19]. The latter studies only considered a very limited complexome and took a simplistic view at differential abundances across cellular states.

Whereas databases of experimentally detected protein complexes continue to serve the community well, they are inherently incomplete - especially when it comes to dynamic combinatorial complexes - and thus can only partially explain all the relevant interplay [20, 21]. Proteins concerned with the regulation of transcription and the chromatin state, for example, are highly interwoven subsets of physically interacting proteins and form complexes in a time-, context- and condition-specific manner. In particular transcription factor complexes are master regulators of all levels of eukaryotic life ranging from the yeast cell cycle [22] to key determinants of cellular fate in mammals [23–25]. We showed with our combinatorial complex prediction algorithm DACO [26] that by integrating connectivity constraints inferred from interactions between protein domains, one is able to unravel the ensemble of biologically feasible protein complexes even for challenging modules of the interactome. With our more recent development PPIXpress [27] and transcript expression data, the input data for DACO can be contextualized to a level of detail that even takes into account potential effects of alternative splicing when inferring sample-specific interactomes.

Here, we present the differential analysis software CompleXChange as a terminal step of a pipeline consisting of PPIXpress-contextualized and DACO-derived protein complexes, or arbitrary alternative input protein complexomes. The tool quantifies protein complexes, includes several statistical testing procedures, is open-source and can easily scale up to 10,000s of interdependent complexes on a standard computer.

## Implementation

**Fig. 1:**
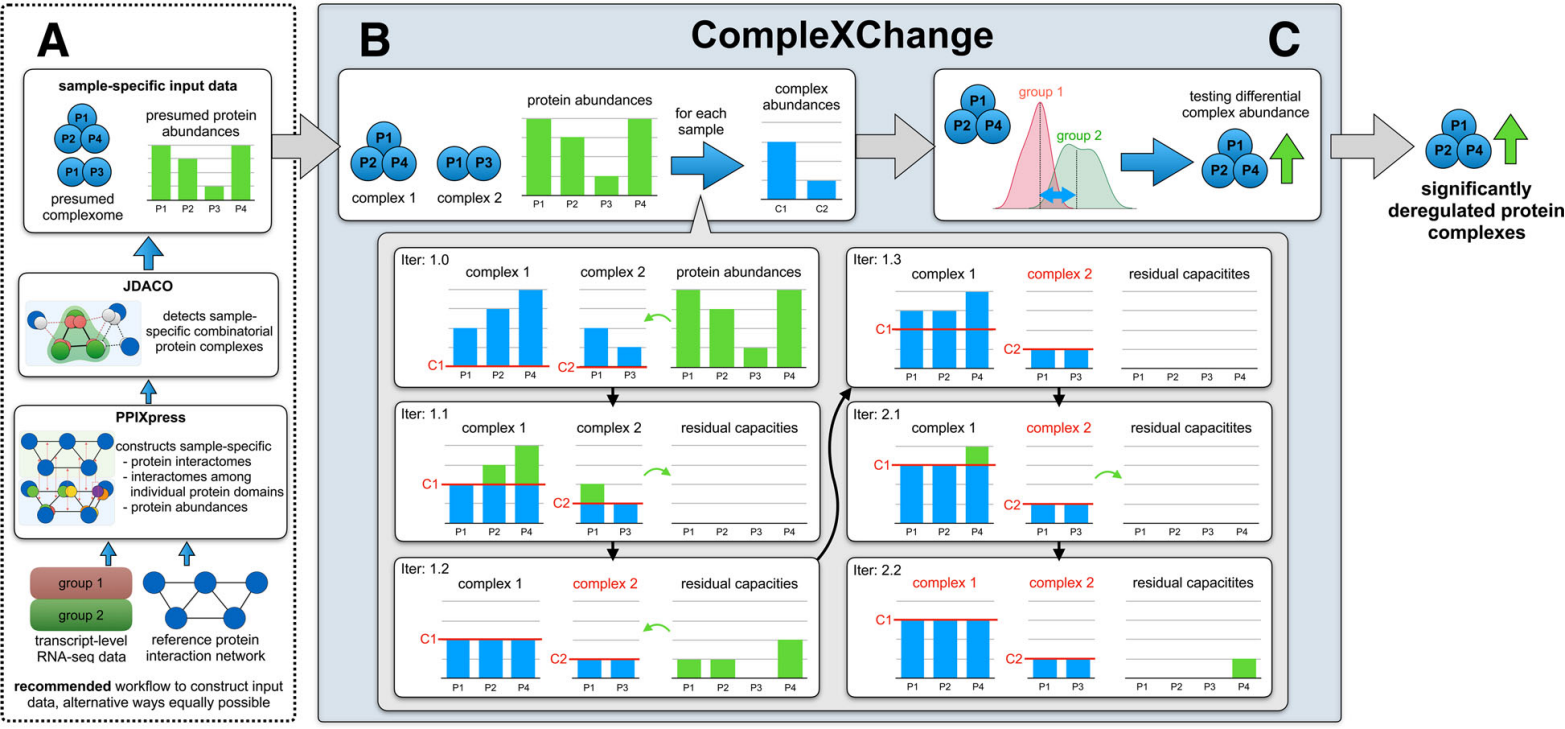
Workflow example for CompleXChange. A) Suitable input data can be constructed easily with either PPIXpress and JDACO or in suitable alternative ways. CompleXChange then performs B) the approximation of complex abundances, and C) the detection of differential complexes. Details are described in the main text

CompleXChange facilitates differential analyses of the protein complexome. It is intended to be used with input data on two groups of samples for which protein complexes and protein abundances are predicted by the tools JDACO [26] (version 1.0+) and PPIXpress [27] (version 1.15+). The software can also be applied to suitable input data from alternative origin, of course. An alternative workflow is provided below on the example of reference complexes taken either from CORUM [28] or from hu.MAP [21]. A ready to use platform-independent Java 8 binary, a user guide with example data and the source code of the program are freely available for download at https://sourceforge.net/projects/complexchange/. The general workflow is outlined in Fig. 1.

### Approximating protein complex abundances

In the first computational step, CompleXChange infers complex abundances from the input data, namely total protein abundances and protein complexes, for each individual sample. To speed up the calculations, CompleXChange automatically utilizes multi-core systems in this step by exploiting the independence of the samples.

Binding affinities between proteins are neglected as suitable data is lacking currently and in the foreseeable future [29]. Instead, we assume that the formation of complexes in a cellular sample is governed by two basic rules: the total amount *p*_*i,tot*_ of each protein *i*∈*P* in the sample is fixed, see Eq. 1, and the abundance *c*_*m*_ of a complex *m*∈*C* is limited by its least abundant member protein, see Eq. 2. Thus

1 $$\begin{array}{@{}rcl@{}}  \forall i \in P: p_{i,tot} = \sum\limits_{m \in C} p_{i, m} + p_{i, res}, \end{array} $$

2 $$\begin{array}{@{}rcl@{}}  \forall m \in C: c_{m} = \min_{i \in C_{m}} p_{i, m} \end{array} $$

where *C*_*m*_ denotes the set of proteins that make up complex *m*, *p*_*i,m*_ is the amount of protein *i* that is assigned to complex *m*, and *p*_*i,res*_ is the residual quantity of protein *i* that is unbound in the sense of the input proteome *P* and complexome *C*. We do not consider the case that single proteins may occur as multiple copies in a protein complex because there are few data available and the information is absent in the notion of complexomes derived from interaction networks. The methodology can in principle be extended to cover stoichiometries of the important class of homo-oligomeric protein complexes if that information should become widely available at genomic scale in the future. At the moment, our concept of neglecting such complexes leads to an over-representation of the other complexes that these proteins are involved in. Figure 1b visualizes an application of the algorithm to an artificial example where “Iter: *x*.*y*” means iteration *x* and step *y*.

#### Step 0: Initial distribution of proteins

The algorithm starts by distributing equal portions of the total abundance of each protein *p*_*i,tot*_ to the complexes it is participating in. Thus ∀*i*∈*P*:*p*_*i,res*_=0 and

3 $$\begin{array}{@{}rcl@{}}  \forall i \in P, m \in P_{i}: p_{i, m} = \frac{p_{i,tot}}{|P_{i}|} \end{array} $$

where *P*_*i*_ is the set of all complexes that include protein *i*. This step is only executed once.

#### Step 1: Tracking surplus capacities

After the initial fill-up in Step 0 and subsequent redistribution steps in later iterations, the limiting proteins in each complex are determined and all *c*_*m*_ are set according to Eq. 2. Thereby, all complexes limited by a protein *i* in this iteration are kept track of in *L*_*i*_. Residual capacities are subsequently updated by the surplus protein amount, thus

4 $$\begin{array}{@{}rcl@{}}  \forall i \in P: p_{i, res} = \sum\limits_{m \in P_{i}} (p_{i, m} - c_{m}). \end{array} $$

The respective quantities per complex are then adjusted accordingly, ∀*i*∈*P,m*∈*P*_*i*_:*p*_*i,m*_=*c*_*m*_.

When its limiting proteins have zero residual capacity at this point, their share in the complex will remain fixed in further iterations and thus the complex is saturated. Saturated complexes and proteins solely found in saturated complexes are therefore set aside and not considered in future iterations (see change to red text color for complex annotations in Fig. 1B).

#### Step 2: Detecting convergence

The sum of residual capacities $\sum \nolimits _{\forall i \in P} p_{i, res}$ after Step 1 is monotonically decreasing and the optimal state is found when no further meaningful decrease is possible. The iterative optimization stops and the complex abundances *c*_*m*_ are returned when either $\Delta \sum \nolimits _{\forall i \in P} p_{i, res} < \epsilon $, the preset maximum number of iterations is reached or all complexes are saturated. Details regarding default termination parameters are given in Suppl. Additional file 1: Section S1.1.

#### Step 3: Redistributing residual capacities

To counteract optimization confinement (pathological examples can be artificially constructed where protein amount is swapped back and forth without any meaningful improvement) and accelerate convergence, a logistic saturation function that is decreasing rapidly with each iteration sets a distribution prefactor *λ*∈[0.99,…,0.09) (see Suppl. Additional file 1: Section S1.1 for details) by which the residual amounts of limiting proteins ({*i*∈*P*||*L*_*i*_|>0}) are preferentially distributed to complexes they limit:

5 $$\begin{array}{@{}rcl@{}}  \forall \{i \in P \bigm| |L_{i}|>0\}, m \in L_{i}: p_{i, m} = p_{i, m} + \frac{\lambda p_{i, res}}{|L_{i}|} \end{array} $$

with thus remaining capacitites ∀{*i*∈*P*||*L*_*i*_|>0}:*p*_*i,res*_=(1−*λ*)*p*_*i,res*_. Finally, the complete residual capacities of all proteins are distributed equally as $\forall i \in P, m \in P_{i}: p_{i, m} = p_{i, m} + \frac {p_{i,res}}{|P_{i}|}$ and therefore ∀*i*∈*P*:*p*_*i,res*_=0. From here, the algorithm proceeds with Step 1 in a new iteration.

### Detection of differential complexes

After annotating each complex detected by JDACO with an abundance value per sample, we statistically evaluate the numerical difference of the abundance of individual complexes between groups (see Fig. 1C). To limit unnecessary testing, complexes that should undergo testing have to be detected in at least a sizeable fraction of samples of either group (default: 0.75). The group-specific distributions of each complex that passed this filtering step are then subjected to two-sided statistical tests. Implemented statistical tests are the Wilcoxon rank sum test (default test; unpaired, non-parametric), Welch’s unequal variances t-test (unpaired, parametric), Wilcoxon signed-rank test (paired, non-parametric), and the paired t-test (paired, parametric). Multiple testing adjustment is subsequently performed using the Benjamini-Hochberg procedure [30] and significantly deregulated complexes are reported. Additional options that are implemented in the code but not discussed here are (a) to base the differential analysis on subsets of complexes detected to help the detection of alterations in robust core complexes, or (b) to solely use combinations of user-specified seed proteins as the reference of interest. Please refer to the user guide for details.

Furthermore, CompleXChange includes an optional analysis that determines seed proteins that occur more often than expected by chance in up- or down-regulated complexes. If this option is selected, a ranked list of protein complexes is constructed by assigning a score to each evaluated complex. This score is set as the negative logarithm of their raw p-value and the sign of their direction of deregulation. In doing so, the task resembles the established approach Gene Set Enrichment Analysis (GSEA) but is applied to proteins in scored protein complexes in an analogous way. The implementation is done according to the original GSEA paper [31]. By default 10,000 iterations are made in the randomization step and only seed proteins are considered that belong to at least 10 complexes.

## Results

The results and their discussion will be divided into three major parts. First, we introduce the datasets that were used in our evaluation, then we assess the performance of CompleXChange. Finally, we analyze the results of an application of CompleXChange to derive the differential transcription factor complexome of classical and non-classical monocytes.

### Datasets and processing of data

#### Preparing sample-specific transcript expression data

Raw RNA-seq data for 17 samples of classical monocytes (CMs) and for 17 samples of non-classical human monocytes (NCMs) [32] (16 sample pairs matched by donor among them) were retrieved from the SRA (accession SRP082682) [33]. A subset of 58 RNA-seq samples of Finnish women among the human lymphoblastoid cell line samples (LCLs) of the GEUVADIS data [34] was downloaded from EBI ArrayExpress (accession E-GEUV-1) [35] analogously to [36]. The raw sequencing data was quantified using kallisto 0.43.1 [37] and the annotation data on human protein-coding transcripts of GENCODE release 27 (GRCh38.p10, Ensembl release 90). Kallisto was applied with bias-correction enabled and default options otherwise. Fragment length estimates for the single-end sequenced monocytes data were set according to the original publication [32]. One hundred iterations of bootstrapping were carried out to account for technical variation in a subsequent differential analysis using sleuth [36].

#### From interaction networks to transcription factor complexomes

From the weighted human protein-protein interaction network PrePPI [38, 39] we downloaded its most recent high-confidence release on 17. Jan 2017. On the basis of this reference interactome we constructed sample-specific protein-protein interaction networks as well as corresponding domain-domain interaction networks for all quantified transcript expression samples with PPIXpress 1.18 [27]. For this, the most recent updates were automatically retrieved from Ensembl (release 90) [40], UniProt (release 2017_09) [41] and 3did (release Sept 2017) [42]. The reference network contained information on 18,451 proteins and 1,527,335 interactions. 70% of the proteins and 37% of the protein interactions were mapped to domain interactions and thus can benefit from the transcript granularity of the data and the methodology of PPIXpress that adapts the interactome in an isoform-specific manner. The usefulness of this model based on conserved domains was recently confirmed experimentally [43, 44]. All transcripts with a non-zero TPM value were deemed expressed. Approximate protein abundances were taken as the sum of TPM values for all expressed transcripts coding for the protein. Notably, when assigning abundance values, PPIXpress (since version 1.12) by default excludes transcripts with Ensembl biotype annotations ’nonsense-mediated decay’ or ’non-stop decay’. Although protein abundances are still approximated by mRNA expression, the pipeline already accounts for well-understood post-translational surveillance mechanisms and should thus provide more reasonable estimates than mere gene expression data until equally rich genome-wide proteome abundance data are available in appropriate sample sizes.

**Fig. 2:**
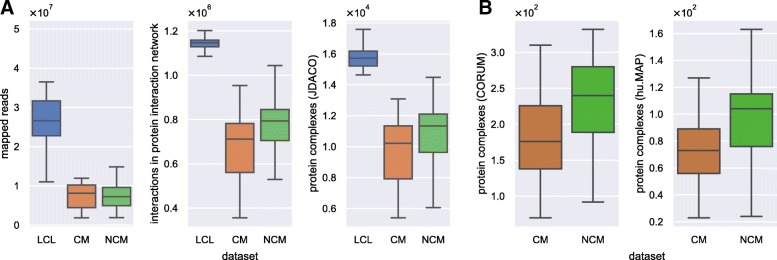
Size distributions of input data. A) Number of mapped reads (left), size distributions of sample-specific protein interaction networks (middle) and JDACO predicted complexomes (right) for individual samples of the GEUVADIS lymphoblast cell line (LCL) data and classical (CM), as well as non-classical monocytes (NCMs). B) Sizes of sample-specific monocyte complexomes derived from the data of CORUM (left) and hu.MAP (right)

Finally, transcription factor (TF) complexes were predicted for each sample with JDACO 1.0 [26] by employing the 601 TFs annotated in HOCOMOCO v10 [45] as seed proteins in the respective protein and domain interactomes. The seed pair threshold was set to 0.95 (PrePPI weights are probabilities), the maximal complex size to 5 proteins (optimized tradeoff between allowed complex size and runtime) and default parameters were used otherwise. The thus derived complexome will serve as the default input for our analyses. Figure 2a visualizes the distributions of mapped reads (left) as well as interactome (middle) and predicted complexome sizes (right) for the three groups of samples used in the study.

To illustrate how CompleXChange can also be used in alternative workflows, we downloaded the manually curated human protein complexome of 2916 complexes in CORUM (3.0) [28] and the precompiled dataset of 4526 hu.MAP complexes [21] which was derived by data integration efforts. After filtering for complexes with at least one TF, the 454 remaining CORUM transcription factor complexes (TFCs) comprised complexes involving 159 TFs. The 277 remaining hu.MAP TFCs covered 183 TFs. The thus derived TFCs of each data source where then used as reference complexomes to construct sample-specific subsets for which all member proteins have a non-zero protein abundance (as given by PPIXpress, see above) in the particular monocyte samples considered here. Figure 2B shows the respective complexome sizes for all monocytes samples. When assessing the sizes of TFCs in the CORUM and hu.MAP data, the vast majority of complexes was within the threshold of 5 proteins per TFC that we used in our predictions (see Additional file 1: Figure S1).

### Assessing the methodology

We first evaluated the performance of the algorithm that approximates protein complex abundances implemented in CompleXChange. For this, we compared the ComplexChange results to an approach where the problem was formulated as a linear program [18, 19]. Simulated data with known ground truth was used to benchmark the two methods. Furthermore, we checked if CompleXChange was susceptible to reporting deregulated complexes erroneously and how it behaved using limited data. To emulate rather complete complexomes, all method evaluation was conducted using the extensive predicted complexomes of each sample.

#### Comparing abundances computed by CompleXChange and linear programming

**Fig. 3:**
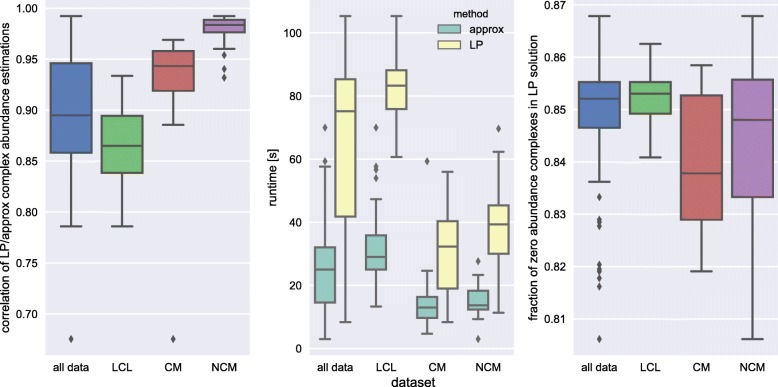
Comparison of complex abundance estimations by the iterative approximation in CompleXChange (approx) and by linear programming (LP). Shown are the correlation of their results (left), the necessary runtimes for each method (middle) and the fraction of zero abundance complexes reported by the LP approach (right) per dataset as well as accumulated for all data. Runtimes were calculated as the average of 3 repetitions for each method and sample

Using both the CompleXChange algorithm and an existing approach based on linear programming (LP) we computed abundance values of predicted protein complexes for all 92 samples on monocytes and lymphoblastoids. The LP approach was implemented according to the equations in [18] using the established open-source solver lpsolve (v5.5) [46]. Figure 3 visualizes the correlation of complex abundance estimation results between both methods (left), runtimes for each method (middle) and the fraction of complexes per sample that were assigned with an abundance of zero by the LP-based approach (right).

The predicted protein complex abundances were overall very similar to each other with an average correlation of 0.90±0.06 (Fig. 3, left). Computing the LP results took on average 2.8±0.9 times longer (Fig. 3, middle) than using CompleXChange on identical input data (*p*<10^−16^, two-sided Wilcoxon signed-rank test paired by sample). Notably, the LP formulation resulted in many zero solutions. On average, 85*%*±1*%* of all complexes in a sample were assigned an abundance of zero (Fig. 3, right) although all member proteins in input complexes have non-zero abundance by definition. Whereas the LP result is numerically optimal given its formulation and constraints, non-sparse abundance results as returned by CompleXChange - even if they are very small - appear biologically more reasonable solutions. Furthermore, zero-inflated complex abundance distributions would require an adjusted statistical treatment [47, 48].

#### Benchmarking complex abundance estimation on simulated data

As pointed out before, there exists so far no adequate experimental reference data to test the complex abundance estimation against. In lieu of this, we generated input data of known ground truth by randomized construction on the basis of realistic complex compositions and expression values from our prepared samples. The construction reverses the simple idea that a protein which is exhaustively incorporated into complexes and has no unbound portion (*p*_*i,res*_=0) consequently sets the maximum abundance of all complexes it is part of. For the construction of this synthetic dataset, the total abundance *p*_*i,tot*_ of each limiting protein is randomly drawn from sample data. To ensure that such a sampling does not suffer from biological bias, we assessed if protein abundances correlate with the number of complexes a protein participates in. In the data on (N)CMs and LCLs this was clearly not the case (average correlation −0.005±0.003). The arbitrary association of proteins with abundance values should therefore be unproblematic.

Distributing a respective share of all limiting proteins to the complexes in which they take part can then be modeled in various ways (model parameter I). All *p*_*i,m*_ are determined by definition (see Eq. 1-2) and only residual capacities *p*_*i,res*_ of non-limiting proteins remain to be set (model parameter II). These in turn specify the *p*_*i,tot*_ of the artificial input data. Model parameter I, the distribution of limiting protein abundances among their associated complexes, was realized using three independent modules: equal distribution with modeled noise (abbreviated as eqd-[noise parameter]), sampled from an empirical distribution (ed) from ComplexChange approximation results and an assumption-free random distribution (rndd). Model parameter II is the unbound ratio parameter that models the extent of residual capacities of non-limiting proteins. The detailed construction schemes as well as our estimates on reasonable noise parameter ranges are documented in Suppl. Additional file 1: Section S1.2.

**Fig. 4:**
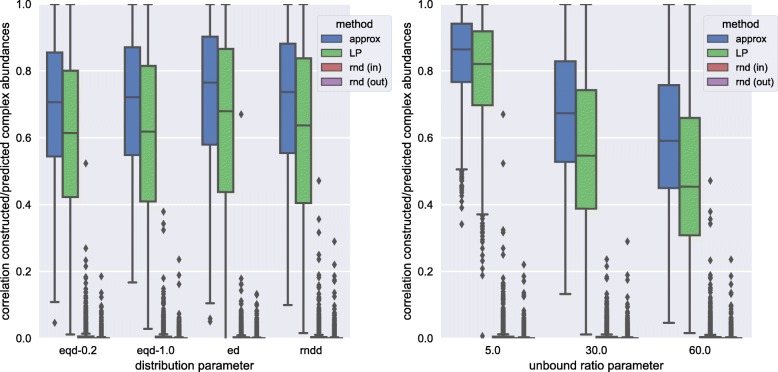
Correlation of constructed complex abundances and predictions by different estimation methods depending on different modeling parameters. Results are shown in dependency of the distribution parameter (left) and the unbound ratio parameter (right)

To judge the relative performance of the CompleXChange approximation algorithm we also applied the LP approach and two randomized modifications of the CompleXChange algorithm to the artificial reference data. In the first randomized variant of the algorithm, input abundance values of proteins were shuffled before applying the abundance estimation method (abbreviated rnd (in)), i.e. input protein abundances did not match the abundance of the proteins associated in the ground truth. In the second variant, the complex abundance results derived from the correct input data were shuffled (abbreviated rnd (out)). We tested 12 combinations of parameters over all 92 samples for 20 iterations each for all methods (see Suppl. Additional file 1: Section S1.2 for details on parameter sets). To assess the smoothness of the CompleXChange approximation performance, a broader set of 42 combinations including some intermediate values was used for benchmarking. We compared the artificial data for which we knew the ground truth with the results of the individual methods in terms of the correlation of known/predicted complex abundances. The results are shown in Fig. 4 in dependency of the distribution parameter (left) and the unbound ratio parameter (right). More detailed results for all individual parameter sets are shown in Additional file 1: Figure S5.

Both CompleXChange approximation and the LP approach performed far better than the randomized methods whose results were generally not correlated with the reference complex abundances (see Fig. 4 and Additional file 1: Figure S5 for details). The correlation of the CompleXChange results with the reference was significantly higher than those from LP across all modeling parameter sets (*p*<10^−14^ for all parameter sets, see Additional file 1: Additional file 1: Table S1 and 1: Figure S5 for details). Interestingly, the performance of both methods was more strongly affected by the unbound ratio (model parameter II) than by the modeling of the distribution of protein product (model parameter I). This is even more apparent when a broader choice of modeling parameters is applied, as was done for the CompleXChange abundance estimation (see Additional file 1: Figure S6). Consequently, a good coverage of the complexome sets the ruleset of interdependency and also limits excess protein product. The typical complexome size in our study (see Fig. 2, rightmost) was about a magnitude larger than, for example, that used in the study describing the application of the LP-based approach to human [19] (1,338 human protein complexes taken into account).

#### Detection of false positives in negative control data

The subset of Finnish women in the GEUVADIS data that we prepared is assumed to be rather homogeneous. Hence, random sampling of groups therein was used before as a negative control in the assessment of differential expression methods [36]. When we analogously applied the same testing approach to find deregulated complexes in the GEUVADIS data, CompleXChange showed a high robustness against false positive reports when group sizes were reasonably balanced. For details, we refer to Suppl. Additional file 1: Section S2.1.

#### Sample size dependency of results

We also checked by subsampling on a reference dataset (see [36, 49, 50]) how CompleXChange behaved when only a small number of samples is available for differential analysis. The results indicated that at least 10 samples per group should be used, if possible. For details, see Suppl. Additional file 1: Section S2.2.

### Differential transcription factor complexome of classical and non-classical monocytes

**Fig. 5:**
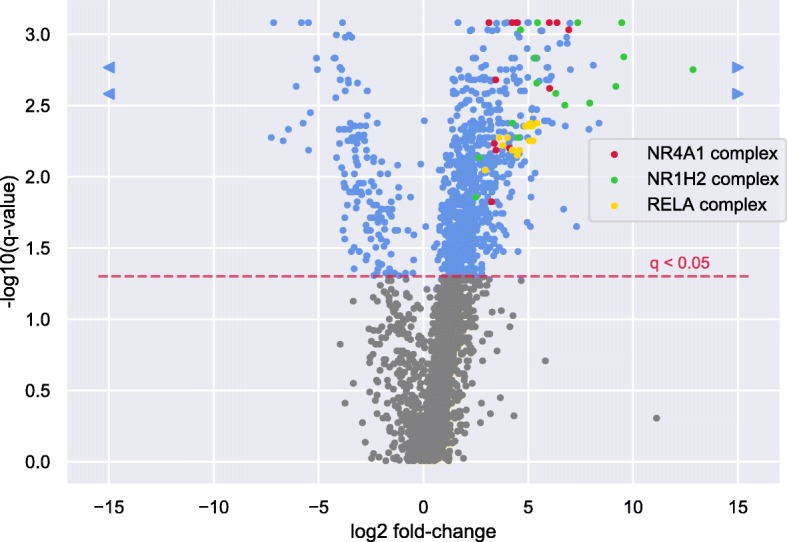
Volcano plot of fold-changes in protein complex abundances. Significantly deregulated complexes between classical and non-classical monocytes are shown as blue points. Complexes below the significance threshold are colored grey. Additionally, complexes that contain one of the three most enriched TFs are shown in red (NR4A1), green (NR1H2) and yellow color (RELA), respectively. Fold-changes were computed as the ratios of mean abundances of respective complexes in the two groups. Complexes that exhibited border case fold-changes (zero mean abundance in one of the groups) were set to ±15 and the respective datapoints marked as triangles

Finally, we applied CompleXChange to detect deregulated TF complexes (TFCs) between classical and non-classical monocytes whereby complexomes were predicted for each sample with PPIXpress and JDACO. This cellular transition was chosen because the expected differences should be comparably small and the number of samples in the dataset appeared sufficient. We used non-parametric testing, FDR 0.05 and default settings otherwise and enabled the option to assess if seed proteins (here: transcription factors) are enriched in up- or down-regulated complexes. CompleXChange reported 978 deregulated TFCs and 35 enriched TFs therein. Figure 5 shows a volcano plot of the complexes evaluated and the distributions of complexes involving the three most enriched TFs.

#### Comparison to differential expression results

Differentially expressed genes were determined using the quantified RNA-seq data (see Materials and Methods) and sleuth (v0.29.0) [36]. For this, transcript expression was summarized to the gene-level using matching Ensembl 90 data retrieved by biomaRt (v2.34.0) [51] and statistical significance was determined based on q-values below 0.05 in likelihood ratio- and Wald-tests. As result, 316 genes were found to be differentially expressed, 77 of those were TFs. In the following, these genes are termed DE genes. We assume that the proteins encoded by them are deregulated as well.

**Fig. 6:**
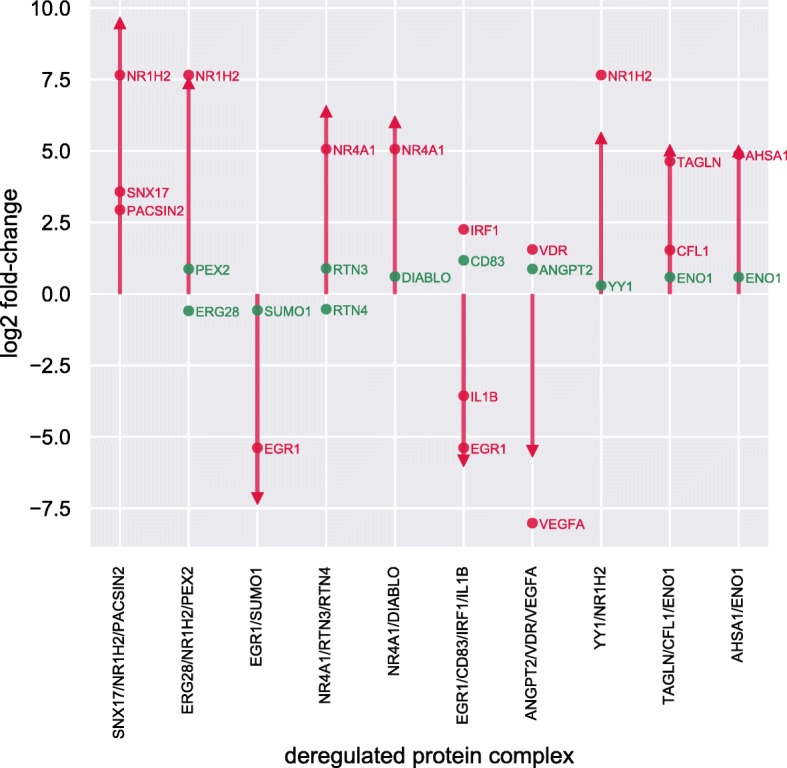
Fold-changes of the top-10 deregulated transcription factor complexes and their members. The 10 most deregulated TFCs are shown in order of significance on the x-axis, the arrowtips on the y-axis depict their logarithmic fold-change. In addition, logarithmic fold-changes of their member proteins are overlayed on the respective columns whereby proteins coded by DE genes are colored red and those with non-DE genes associated are shown in green. Fold-changes were computed as the ratios of mean abundances of respective complexes and proteins in the two groups

We first studied to what extent DE genes overlapped with the 978 deregulated complexes. On average, about a third (37*%*±24*%*) of each reported deregulated complex consisted of proteins whose genes were deregulated between the two cell types. In 823 complexes (84.2% of all results) at least one protein-coding gene was deregulated and in 32 cases (3.3%) all were differentially expressed. These modes of action were also relevant in the 10 most deregulated complexes as can be seen in Fig. 6. The significantly altered abundance of 155 complexes (15.8%) could not be inferred by differential expression analysis of protein-coding genes in isolation. Such events can be explained, on the one hand, by the effects of mutual dependence among the complexes since they share and compete for each protein product, and, on the other hand, by the dynamics of the neighborhood in the protein interactome which affect the cohesiveness measure in JDACO predictions (see [26]).

Next, we investigated the relationship between deregulated complexes and deregulated protein-coding genes in reverse direction. Of all 87,945 complexes seen in any sample 54,645 had at least one deregulated gene (62.1%). Among those complexes only 1.5% were detected as being deregulated. When complexes were filtered with CompleXChange to be present in at least 75% of either group, of the 2,522 complexes, 1,841 had a deregulated member (73.0%). Only 44.7% of those complexes were found to be deregulated by CompleXChange. We stored the 1,841 complexes selected by DE for the analysis of information content in the next subsection.

At last, we compared the results in terms of TFs that were reported as deregulated. Whereas most of the TFs that were found to be enriched in deregulated complexes were also differentially expressed, 14 of the 35 (40%) enriched TFs were not significantly deregulated on the gene-level. Significance rankings between the two approaches can not be compared, as can be seen in Additional file 1: Table S2. Most noteworthy, NR4A1 is the TF with highest enrichment in CompleXChange, whereas in DE it is only the eighth TF when sorting by q-value and the 22nd TF when sorting by fold-change. Nr4a1 is the master regulator of non-classical monocytes in mice [52, 53]. In human, its ortholog NR4A1 is assumed to have the same regulatory function [54, 55].

Furthermore, DE TFs and TFs reported to be enriched in deregulated complexes were subjected to overrepresentation analyses using the webservices GeneTrail2 [56] and PANTHER [57] against the background of all 601 TFs regarding five pathway annotation databases. Details concerning the analyses and the complete results are provided in Suppl. Additional file 1 Section S2.3. Whereas the DE TFs showed only one rather unspecific enriched term in one database (PANTHER pathway “CCKR signaling map” 3.54-fold enriched, see Additional file 1: Table S3), the CompleXChange enriched TFs showed enrichment for all pathway annotation databases. The enriched annotations contained, for example, Toll(-like) receptor signaling (several terms across databases, see Additional file 1: Tables S5, S7 and S8), TNF(- *α*) signaling (several terms across databases, see Additional file 1: Tables S5 and S7), various specific interleukin signaling pathways (e.g. WikiPathways “IL-1 signaling pathway", 13.71-fold enriched, see Additional file 1: Table S7) as well as more general terms such as “Inflammation mediated by chemokine and cytokine signaling pathway” (PANTHER pathway, 9.95-fold enriched, see Additional file 1: Table S8). This matches the specialization that has been reported for these cell types [58–60].

#### Comparison to an alternative pipeline using CORUM and hu.MAP complexomes

**Fig. 7:**
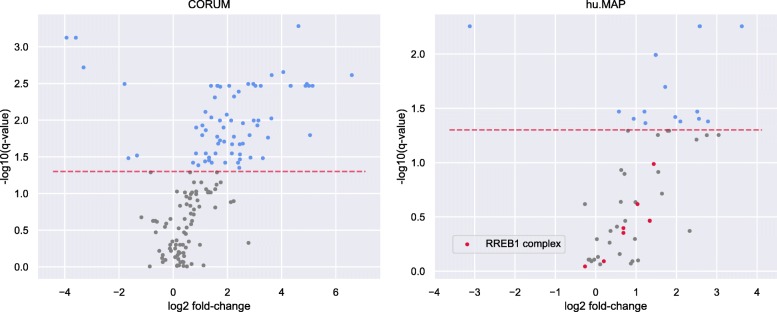
Volcano plots of fold-changes in CORUM and hu.MAP protein complex abundances. The results for CORUM complexes are shown in the left plot and the results on the basis of hu.MAP complexes on the right. Significantly deregulated complexes between classical and non-classical monocytes are depicted as blue points and complexes below the significance threshold are colored grey. For the hu.MAP results, complexes that contain RREB1 are shown in red. Fold-changes were computed as the ratios of mean abundances of respective complexes in the two groups

For this comparison, we selected those protein complexes of CORUM and hu.MAP containing at least one TF instead of using predicted complexomes as the input. When we applied CompleXChange to the sample-specific subsets of the CORUM and hu.MAP complexomes using the same parameters as with the JDACO predictions, 77 CORUM complexes and 16 complexes of hu.MAP were identified as deregulated between classical and non-classical monocytes. The distribution of complex abundance changes appeared very one-sided in both datasets, see Fig. 7. Due to the very small number of complexes assessed and due to the skewed distributions, the calculation of TFs enriched on the upper/lower end of the deregulation range is not really meaningful. This can be seen on the example of RREB1 that was found to be the only enriched TF for the hu.MAP data.

The overlap between results of CompleXChange analyses using the predicted JDACO complexes and complexomes of CORUM and hu.MAP was overall very small (see Table 1). Interestingly, the result derived from the predicted sample-specific complexomes was more similar to either result of the two complex databases than the overlap of the two databases (first three rows, Table 1). This also holds true when all TFCs are taken into account rather than only the ones deemed significant by CompleXChange (last row, Table 1). This is not very surprising since current experimentally-backed complexome libraries are still considered to be quite incomplete [20, 21]. Especially when taking into account the important interplay between complexes sharing proteins, input data of predicted complexomes seem more appropriate in this specific issue.

**Table 1:** Comparing deregulated complexes of JDACO, CORUM and hu.MAP

Results sets compared	Exact matches	Average overlap	Reasonable overlap [%]
CORUM / JDACO	0	0.24±0.12	32.5
hu.MAP / JDACO	0	0.17±0.16	18.8
hu.MAP / CORUM	1	0.08±0.24	6.3
hu.MAP (all) / CORUM (all)	5	0.12±0.19	11.9

“hu.MAP (all)” and “CORUM (all)” depict the sets of all TFCs in the respective datasets whereas all other sets cover the reported deregulated complexes. Overlap between two protein complexes was quantified using the overlap score *ω* [66], “average overlap” between two result sets means the average of all best matches in terms of *ω* between the first (smaller) and second (larger) set of reported deregulated protein complexes. The percentage of complexes in the first (smaller) set with any reasonable match (*ω*>0.25, as in [26, 67, 68]) in the second (larger) set is termed “reasonable overlap”

#### Abundances of reported deregulated complexes are meaningful descriptors of cell type

To assess the information content of deregulated complexes in an unbiased way, we tested their ability to act as descriptors in simple random forest models [61] that were trained to classify the monocyte data into classical and non-classical samples.

For each set of complexes (or TFs) tested we performed 100 iterations of stratified 10-fold cross-validation (CV) to account for randomness in dataset partitioning and tree building [61, 62]. The corresponding abundance values of complexes or TFs were used as the features in a random forest classifier with 32 trees (sufficient according to [63]). The number of features considered in each tree split was automatically set to the square root of the number of total input features according to the heuristic established by [64]. Other parameters were kept at the default setting as implemented in scikit-learn (v0.16) [65]. The performance for a set of complexes (or TFs) was then reported as the mean accuracy over all cross-validation iterations. We considered the following cases: (1) all complexes reported by CompleXChange applied to both predicted and reference complexomes, (2) two stricter sets for which we pruned the result for the predicted complexomes by demanding tighter q-values (*q*<0.01 and *q*<0.001), (3) permutation tests where we sampled random complex sets as well as for (4) DE complexes (complexes with at least one DE protein-coding gene associated, see previous subsection), and (5) DE/all TFs (using protein abundances). The results are summarized in Table 2.

**Table 2:** Cross-validation (CV) accuracies of feature sets examined

feature set	set size	CV accuracy [%]
sign. dereg. complexes (*q*<0.05)	978	96.4±1.5
random complexes	978	86.6±2.2
random complexes (filtered)	978	94.2±0.7
sign. dereg. complexes (*q*<0.01)	429	96.6±1.1
random complexes	429	84.3±3.4
random complexes (filtered)	429	93.5±1.2
sign. dereg. complexes (*q*<0.001)	31	97.0±0.4
random complexes	31	66.8±9.9
random complexes (filtered)	31	88.4±3.9
sign. dereg. CORUM complexes	77	93.6±2.6
sign. dereg. hu.MAP complexes	16	85.5±3.9
DE complexes	1841	96.3±1.5
DE TFs	77	96.2±1.4
all TFs	601	94.9±2.0

For the randomized complexes CV accuracy is reported as the mean across all permutations and its standard deviation. For all other evaluated sets CV accuracy depicts the mean and variance for the 100 iterations of CV

The complexes reported by CompleXChange when applied to the predicted complexomes showed monotonically increasing mean accuracy and decreasing variance with increasing stringency and thus decreasing set size (from 978 to 31, compare “sign. dereg. complexes” entries in Table 2). Whereas the significantly deregulated complexes with highest stringency gave the best overall accuracy of all feature sets tested, including DE TFs, most non-random feature sets basically showed similar performance within their standard deviations. The significantly deregulated complexes reported on the basis of the fixed protein complex datasets gave the lowest classification performance of non-randomized descriptors (compare non-randomized entries in Table 2). This strengthens the assumption that predicted complexomes are favorable currently.

**Fig. 8:**
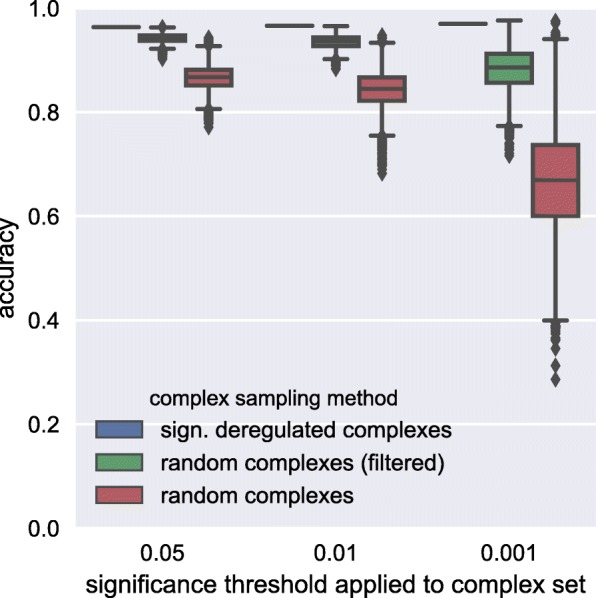
Comparison of CompleXChange results of varying stringency with randomly selected deregulated complexes of equivalent size. Comparison of CompleXChange results of varying stringency with randomly selected deregulated complexes of equivalent size in terms of information content

As a baseline comparison to the CompleXChange results for the predicted complexomes of varying stringency, we evaluated how likely it is to get a similar performance by chance. For this, we drew 10,000 random complex sets of equal size from either all 87,945 predicted complexes seen in any sample or the filtered set of 2,522 complexes. In all tested scenarios, the accuracy of the corresponding CompleXChange result set was very unlikely to be achieved or exceeded by chance (all *p*≤0.0003, see Additional file 1: Table S9 for statistics, Table 2 for averages and Fig. 8 for observed distributions). The hu.MAP-derived deregulated complexes, on the other hand, were often even less predictive than random complexes on average which again encourages to employ complex prediction in this workflow (compare ”sign. dereg. hu.MAP complexes” and all “random complexes" entries in Table 2).

## Discussion

CompleXChange is a new platform-independent open-source tool that enables the quantitative differential analysis of protein complexes derived from sample-specific input protein complexomes and corresponding transcriptome or proteome abundances. To achieve this, our software implements both a fast algorithm to approximate numerical abundances for the protein complexomes found in each sample and a differential analysis that statistically compares the inferred complex abundances between samples of two comparison groups. It thus represents a stand-alone solution for differential analysis of protein complexomes that can be used for large problem sizes. We chose to provide the software as a command-line tool to simplify its application in pipelined workflows.

In the benchmarks detailed earlier we showed that our approach gave better and biologically more reasonable results than an alternative implementation of complex abundance estimation by linear programming while running in a fraction of the time. To demonstrate an application of CompleXChange on a practical example, we presented a case study on human monocyte subsets. In this case study we also showed how profoundly different input data workflows can be realized with CompleXChange, for example using predicted complexomes or by employing reference complexome data. As discussed above, some key findings were supported by the existing literature and further potentially interesting candidates were detected that warrant future experimental studies. Notably, by comparing the CompleXChange results against those from a differential gene expression analysis on the same input data we illustrate that valuable additional information can be gained from the analysis of differential complexes.

CompleXChange currently ignores the binding affinities between interaction partners or exact complex stoichiometries. We agree that these are possible limitations of the approach. However, these are practical considerations made due to the current lack of suitable experimental data on an appropriate scale. The methodology, algorithms and licensing allow for a simple adaption and extension of the software if the according data should become widely available in the future.

## Conclusion

The increasing wealth of transcriptomic data and recently introduced computational tools enable to infer protein interactomes and complexomes in specific samples. With CompleXChange this information can be exploited to conduct differential analyses of the dynamic protein complexome in a quantitative manner. We showed for simulated data with known ground truth that its inferred complex abundances were in better agreement with the artificial reference and made more sense biologically than the runtime-intense mathematical optimization with linear programming. When tested in a realistic scenario, CompleXChange featured a performance regarding robustness and limited amounts of samples that is well-suitable for practical applications. Moreover, reported complexes held significant information content on cellular identity and partially orthogonal information to gene- and protein-centric analyses, which are not covering the physical interplay found in a cell. Hence, analysis of differential complexomes should become even more valuable in the future.

## Availability and requirements

**Project name:** CompleXChange

**Project home page:** https://sourceforge.net/projects/complexchange/

**Operating system(s):** Platform independent.

**Programming language:** Java.

**Other requirements:** Java 8 or higher.

**License:** GNU GPLv3.

**Any restrictions to use by non-academics:** None.

## Additional file

Additional file 1 This PDF contains supplementary text, Figures S1-S11 and Tables S1-S9 that are not included in the main text.

## Abbreviations

CM: classical monocytes
CV: cross-validation
GSEA: Gene Set Enrichment Analysis
LCL: lymphoblastoid cell line sample
LP: linear programming
NCM: non-classical human monocytes
TF: transcription factor
TFC: transcription factor complex
