# Supplementary material for: Differential analysis of combinatorial protein complexes with CompleXChange
Source: BMC Bioinformatics. 2019 Jun 3;20:300. doi: 10.1186/s12859-019-2852-z (PMC6547514; doi:10.1186/s12859-019-2852-z)
Supplement: Supplementary file 1 — This PDF contains supplementary text, Figures S1-S11 and Tables S1-S9 that are not included in the main text. [file 12859_2019_2852_MOESM1_ESM.pdf]

CompleXChange
Supplementary Material

T.Will and V.Helms

S1 Supplementary Methods

S1.1 Approximating protein complex abundances

Default termination parameters

The convergence limit (see Step 2 of the algorithm) was set to $\epsilon = 10^{-7} * |P|$. $|P|$ is the size of the considered proteome P. Thus, ϵ is clearly below the dynamic range attributed to RNA-seq (at least 5 magnitudes [1, 2]) from which the abundance estimates are derived. The maximum number of iterations was set to 10,000.

Saturation function

The distribution prefactor $\lambda \in [0.99, \ldots, 0.09)$ (see Step 3 of the algorithm) is set according to the monotonically decreasing logistic saturation function

$$\lambda(n) = 1.89 - \frac{1.8}{1 + \exp^{-0.05n}}$$

where n is the current iteration (starting with $n = 1$).

S1.2 Constructing artificial data with known ground truth from real samples

The simulation algorithm takes as input a proper sample such as CompleX-Change would use it (protein complex and abundance data) and modeling parameters (see Fig. S2A, complexes C1, C2, C3 and proteins P1, . . . , P5). For constructing the artificial data, the actual mapping of proteins to abundance values is not used. Therefore only the sole abundance values are read. The order of both complexes and abundance values are shuffled in each iteration to randomize the sampling of proteins and complexes.

Now we construct a hypothetical exact state. First, one limiting protein per complex is chosen randomly if no limiting protein is part of the complex yet. Each limiting protein is then associated with any of the original abundance values of the sample (see Fig. S2A). We checked in our data if the abundance value

of a protein is somehow correlated with the number of complexes it participates in to ensure that there is no biological bias in that regard that would be ignored by this random association (see Results Section of the main paper).

Next, the overall abundance of each limiting protein needs to be distributed to all complexes it confines (model parameter I). We implemented three models to do so based on either equal distribution plus noise (see Section S1.2.1), empirical distributions derived from complex abundance approximation results of real samples (see Section S1.2.2), or random partitioning (see Section S1.2.3). See respective Sections of the main text for details.

Finally, artificial abundance values of the proteins need to be set in a meaningful relation to the artificial complex abundances (model parameter II). By the way of construction, limiting proteins have no residual abundance left. Thus their abundance value is exactly the one initially associated with it that is now entirely distributed to the complexes. All other proteins have a larger abundance value than the sum of the abundances of all complexes they participate in. We model this by an unbound ratio parameter r which increases the minimum abundance value $p_{i,base}$ of a non-limiting protein by a relative fraction of $p_{i,base}$ that is sampled from $U(0,r)$, thus $\forall i : p_i = p_{i,base} + p_{i,base}U(0,r)$. The constructed p_i values and the considered complexome are then provided to the complex abundance approximation algorithm and its predicted complex abundances are subsequently compared to the constructed c_i.

For all abundance estimation approaches assessed, we studied the effects of varying the deviation from equal distribution parameters d in the range $\{0.2, 1.0\}$ as well as the empirical and random distributions to cover model parameter I, and unbound ratio parameter r in the range of $\{5, 30, 60\}$ to cover model parameter II. For the CompleXChange estimation a broader range of parameters $d : \{0.2, 0.5, 0.75, 1.0\}$ and $r : \{5, 10, 20, 30, 40, 50, 60\}$ was assessed. The noise parameters were based on the distributions of respective model values seen in the results obtained with CompleXChange from the abundance estimation for the real biological samples. See Section S1.2.4 for details.

Fig. S3 shows the resulting distributions of respective relative deviations and unbound ratios in the constructed artificial data for the different modeling parameter sets that match the range of values that were found in the biological samples. The observed variations were fairly small.

S1.2.1 Distributing abundances to complexes: equally distributed plus noise (abbreviated eqd)

In this scenario, the overall abundance of each limiting protein is distributed in equally sized portions c_{eq} to all complexes it constrains (see Fig. S2A/B). This is equivalent to a situation in which proteins would be distributed if binding kinetics would not matter and all aggregation processes would be equally likely. Whereas this is certainly not the case in reality, at this scale this is the best we can do at the moment [3]. To introduce tractable alterations in the equal partitioning of this abundance c_{eq} per limited complex, we introduce a "deviation from equal distribution" parameter, here called d, which sets the

boundaries of a uniform noise sampling. Each of the formerly equal portions is then shifted by a relative fraction of c_{eq} that is sampled from $U(-d,+d)$, thus $\forall i : c_i = c_{eq} + c_{eq}U(-d,+d)$ (see Fig. S2B). We did not consider a normal distribution here as it is unbounded and may cause a large number of iterations until a valid distribution is obtained.

After shuffling the allocation, the portions are normalized in the sense that their sum is again equal to the protein amount that was distributed initially (see Fig. S2C). If there are non-positive portions, the shuffling process is repeated from step A (see Fig. S2). Finally, the abundances of the complexes associated with the limiting protein that is processed are set to a portion of abundance c_i each (see Fig. S2D). Those are then the artificial complex abundances that are considered as being the ground truth.

S1.2.2 Distributing abundances to complexes: empirical realistic distributions (abbreviated ed)

The second scenario bases the expected deviations for the approximated complex abundances on empirical data using the distribution of limiting proteins to their complexes in the biological data which we considered (see Section S1.2.4). Namely, we analyzed how the overall input abundance of a limiting protein is spread among the complexes of which it is a member. The information is then stored in a map relating the number of complexes that the protein was distributed to and a list of detected partitionings. This map can then be accessed to sample from empirical distributions when constructing artificial samples with ground truth. When there is no entry for a limiting protein affecting m complexes, results for $m_1 = \frac{m}{2}$ and $m_2 = m - m_1$ are queried recursively and concatenated whereby the interval sizes they store are halved to ensure a proper partitioning.

S1.2.3 Distributing abundances to complexes: random partitioning (abbreviated rndd)

In the third scenario, the total abundance of the limiting protein is partitioned randomly by drawing from a uniform distribution and shared accordingly between the complexes.

S1.2.4 Guessing reasonable parameter ranges

Using CompleXChange, abundance values of predicted complexes were derived for all 92 samples of (N)CMs and LCLs as described in the main paper. By summarizing respective complex abundances and comparing them to the input protein abundances, we recovered the residual abundances of all proteins covered by the data. A protein was treated as a limiting protein if its residual abundance was equal or below the limiting threshold 10^{-7} (see Section S1.1). For limiting proteins it was noted how their total abundance was distributed to all complexes they participated in (for the empirical distribution model) and also the average relative deviation from equal distribution (average of $\frac{|c_{eq}-c_i|}{c_{eq}}$ for all affected

complexes c_i). For non-limiting proteins the unbound ratio was noted. For both measures, medians over all proteins were reported per sample.

Fig. S4 visualizes the distribution of the two relevant noise parameters. The relative deviation from an equal distribution of complexes is shown in the left plot and the unbound ratio in the right plot.

S2 Supplementary Results

S2.1 Detection of false positives in negative control data

We conducted a testing approach analogous to [4] and randomly drew two comparison groups of 40, 30, 20, 10 and 5 samples from the homogeneous GEUVADIS-subset consisting of 58 samples. Only group sizes that allowed for sampling without replacement were used. Each combination of group sizes was sampled 5,000 times and CompleXChange was applied using either non-parametric or parametric unpaired statistical testing, FDR 0.05 and default settings otherwise to detect deregulated complexes in the predicted complexomes. Note that due to the randomized selection of two "fake" groups, we did not expect to find any imbalance of protein complexes. Every "hit" can thus be considered as a false positive. Fig. S7 summarizes the results.

Only with very unbalanced group sizes and parametric testing, a small number of false positive complexes was reported regularly (see Fig. S7A: median of 26 reported complexes in 40/5 samples (parametric, see lower plot), 11 in 30/5 (parametric), 2 in 20/5 (parametric), all others median of zero). When non-parametric testing is used, even in the worst case (30/20 samples) less than 1.4% of the runs produced any deregulated complexes (see Fig. S7B, upper left plot) and even less when considering at least 10/100 complexes (see Fig. S7B, upper two rightmost plots). Parametric testing reported almost always some complexes when very unbalanced group sizes (e.g. 40/5 samples per group) were used (see Fig. S7B, lower left plot), often even more than 100 false positive deregulated complexes were detected (see Fig. S7B, lower rightmost plot). Given the increase in statistical power by parametric testing, such a behavior can be anticipated if the emphasis is shifted towards the detection of the differences between individual subsamples rather than the general differences over the whole sample group which should be negligible here. When groups were more balanced, the fraction dropped considerably (see Fig. S7B, lower plots).

S2.2 Sample size dependency of results

By comparing the results of differential analyses on a full dataset with results for random subsets of the data we then investigated how CompleXChange behaved with decreasing amounts of data. For this, we applied CompleXChange to the full data on predicted complexomes of classical and non-classical monocytes and to subsets thereof with different options regarding the statistical testing, FDR 0.05 and default parameters otherwise. We deployed the method to 5,000 random subsets of each combination of group sizes with 15, 10 and 5 samples and to the full data of 17 samples per group assuming unpaired data. Likewise, we ran the tool on the full set of 16 sample pairs matched by donor as well as 500 random subsets of 13, 10 and 5 sample pairs, respectively. All analyses were conducted using both non-parametric and parametric testing. For the result of each individual calculation we computed the precision (fraction of reference deregulated complexes among all reported deregulated complexes when Com-

pleXChange is applied to the subset) and the recall (fraction of reference deregulated complexes found). The F-score is the harmonic mean of precision and recall. The individual results for unpaired/paired non-parametric/parametric testing are shown in Fig. S8-S11.

Unsurprisingly, more samples and balanced group sizes generally improved the agreement between the results for subsets and full dataset. Considering only the results for unpaired testing, the F-score for 15/10 samples in the non-parametric test case was still acceptable with a median F-score around 65% (Fig. S8, upper left plot), whereas the results for parametric testing already broke down severely (Fig. S9, upper left plot). Interestingly, for unpaired non-parametric testing, the decrease in performance up to 10 samples per group was a problem of the recall (Fig. S8, lower right plot), while the precision was actually still reasonably good (Fig. S8, lower left plot). Thus, although CompleXChange may already miss some deregulated complexes if fewer data are available, it is reassuring that, at least, only a comparably small amount of false positives should be reported. When unpaired data is tested parametrically, the statistical power is increased by the assumption of (in our case) a normal distribution of the data. In the case of limited amounts of data, the individual differences between subsamples can be overemphasized compared to the actual differences over the groups as a whole. Consequently, the precision varies strongly even with 10 samples per group, as can be seen in our evaluations (Fig. S9, lower left plot) and the recall drops even further than in the non-parametric case (Fig. S9, lower right plot).

When matched data is available and paired testing can be applied, basically the same observations can be made as for the unpaired data (see Fig. S10 and Fig. S11).

S2.3 Pathway annotation enrichment analyses on transcription factor sets

Both differentially expressed TFs and TFs reported to be enriched in deregulated complexes were subjected to pathway enrichment analyses. For this, we used the webservice GeneTrail2 (version 1.6) [5] with pathway annotation data from BioCarta, KEGG, Reactome and WikiPathways. To complement the analysis, we additionally employed the webservice PANTHER (version 13.1) [6] using their own curated annotation data on PANTHER pathways. For both webservices the background for statistical testing was set to the 601 TFs that were used as seed proteins and respective default parameters for overrepresentation analysis with multiple hypothesis correction otherwise. All individual enrichment results that reported any enriched pathway annotations ($q < 0.05$) are listed in Tables S3-S8.

S3 Supplementary Figures

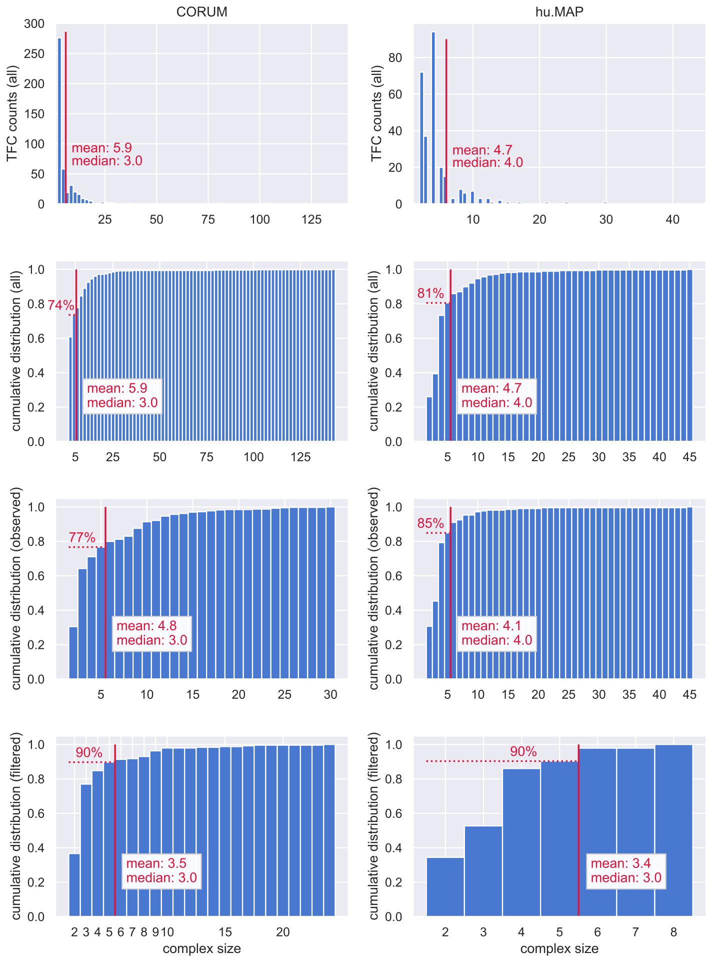

Figure S1: *Size distributions of human transcription factor complexes (TFCs) in CORUM (plots on the left side) and hu.MAP (plots on the right side). The first row shows the counts for different numbers of proteins per TFC (abbreviated "all"). The second row shows the cumulative distributions of TFC sizes for all TFCs in the respective dataset (abbreviated "all"), in the third row for all TFCs observed in the contextualized monocyte samples (abbreviated "observed") and in the fourth row for all TFCs that are observed in at least 75% of samples of either classical or non-classical monocytes as relevant in the differential analysis (abbreviated "filtered"). Additionally given are the percentage of TFCs with at most 5 member proteins as well as mean and median TFC sizes for the respective complexomes.*

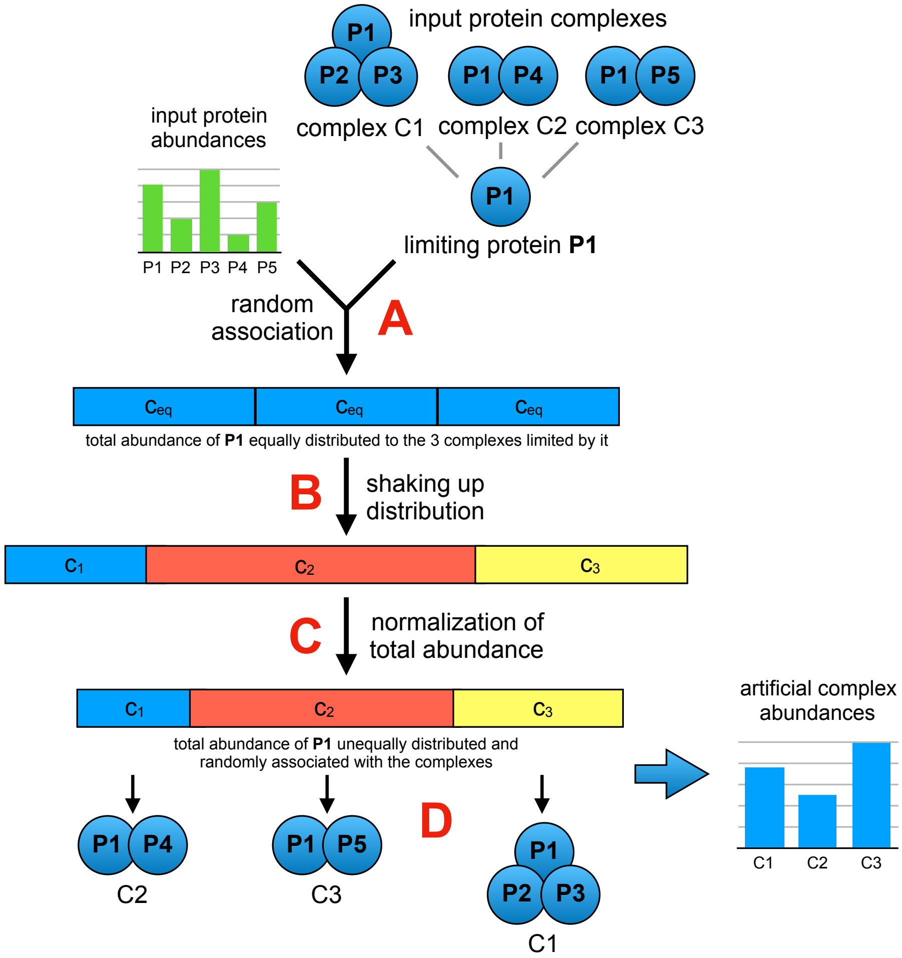

Figure S2: *Constructing artificial complex abundance data with known ground truth from real samples assuming equal distribution of protein product plus noise.*

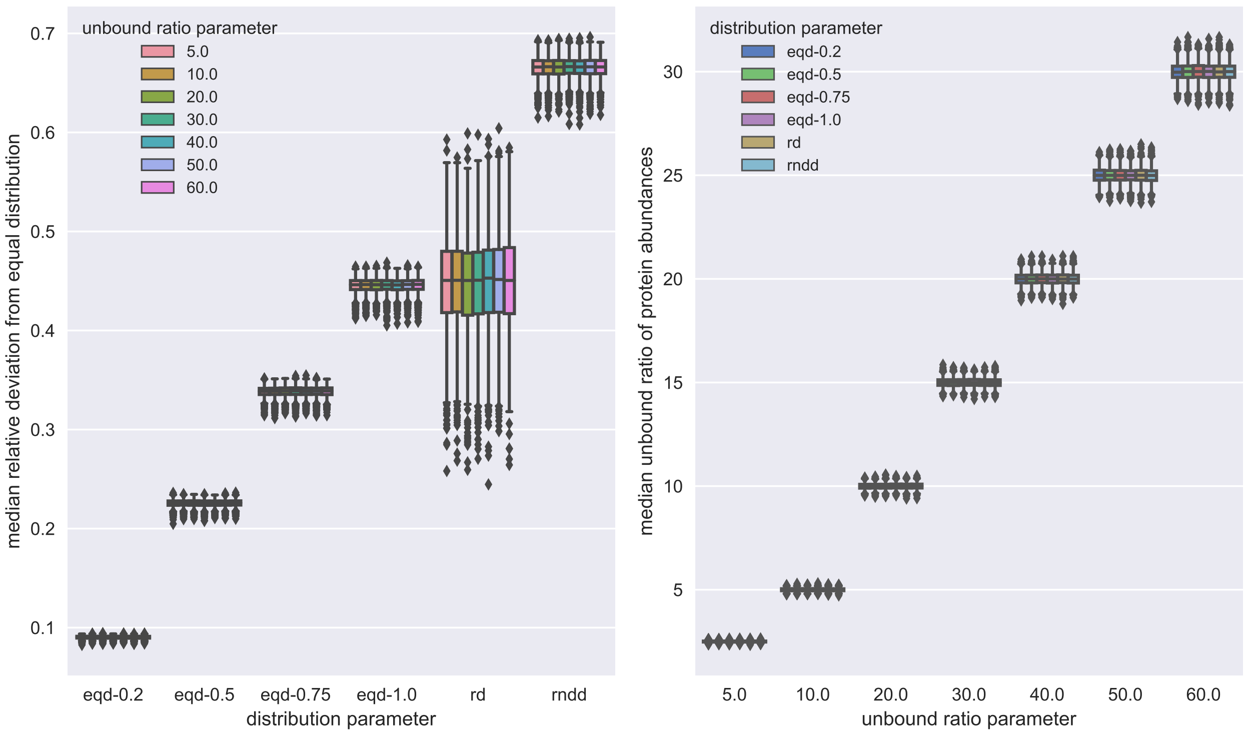

Figure S3: *Parameter ranges of the two relevant noise parameters of the simulation model as found in artificially constructed samples with certain modeling parameter sets (see Section S1.2). The relative deviation from equal distribution of complexes confined by a limiting protein is shown in the left plot and the unbound ratio of protein abundances in the right plot.*

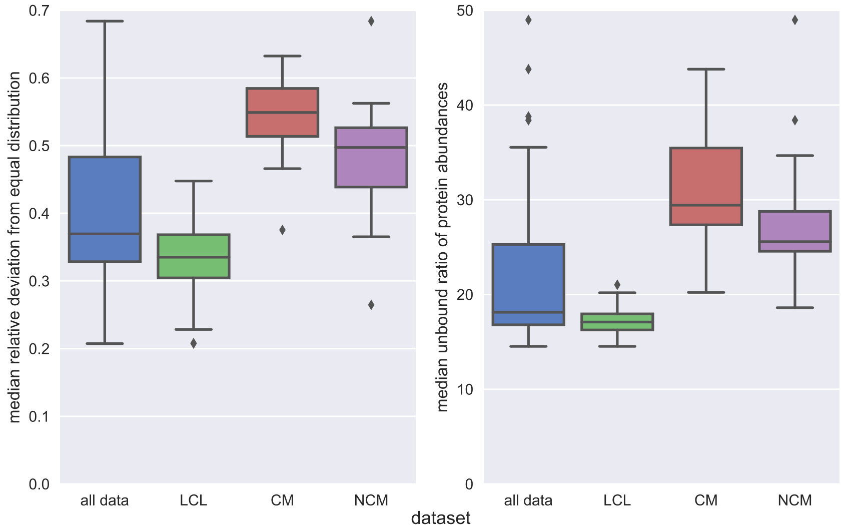

Figure S4: *Parameter ranges of the two relevant noise parameters of the simulation model as found in CompleXChange results for biological samples. The relative deviation from equal distribution of complexes confined by a limiting protein is shown in the left plot and the unbound ratio of protein abundances in the right plot.*

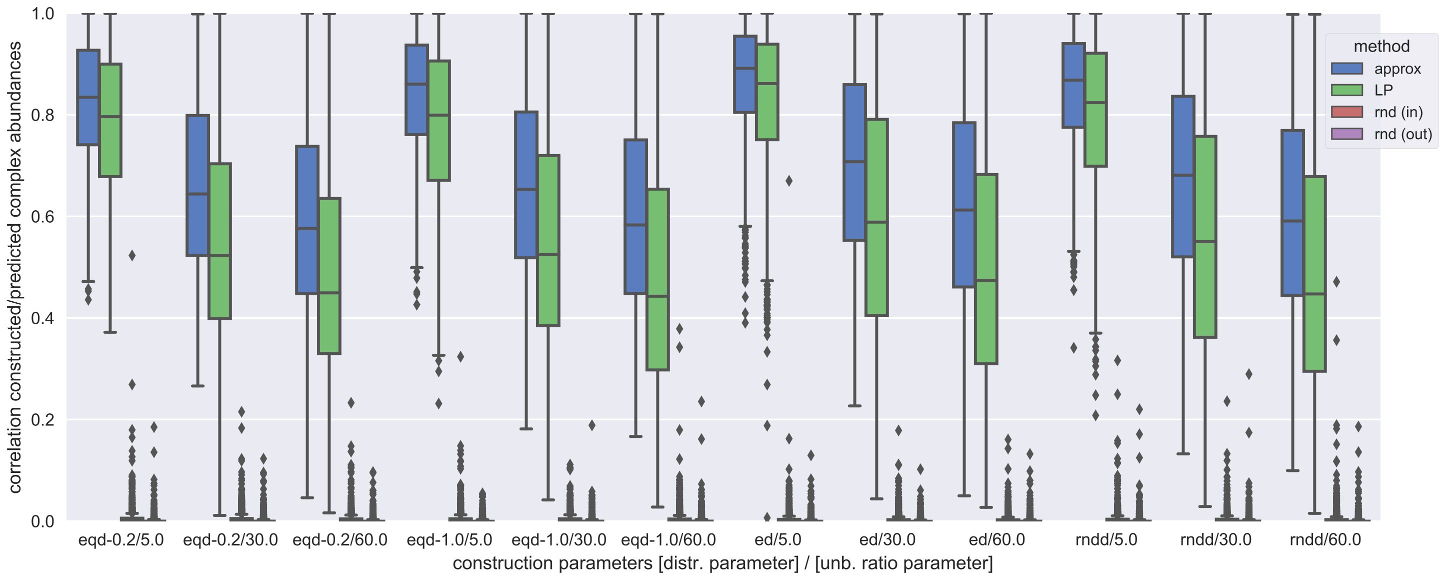

Figure S5: *Correlation of constructed complex abundances in the hypothetical exact state and predictions by different estimation methods depending on different modeling parameters. The results for each parameter set [distribution parameter] / [unbound ratio parameter] are shown individually.*

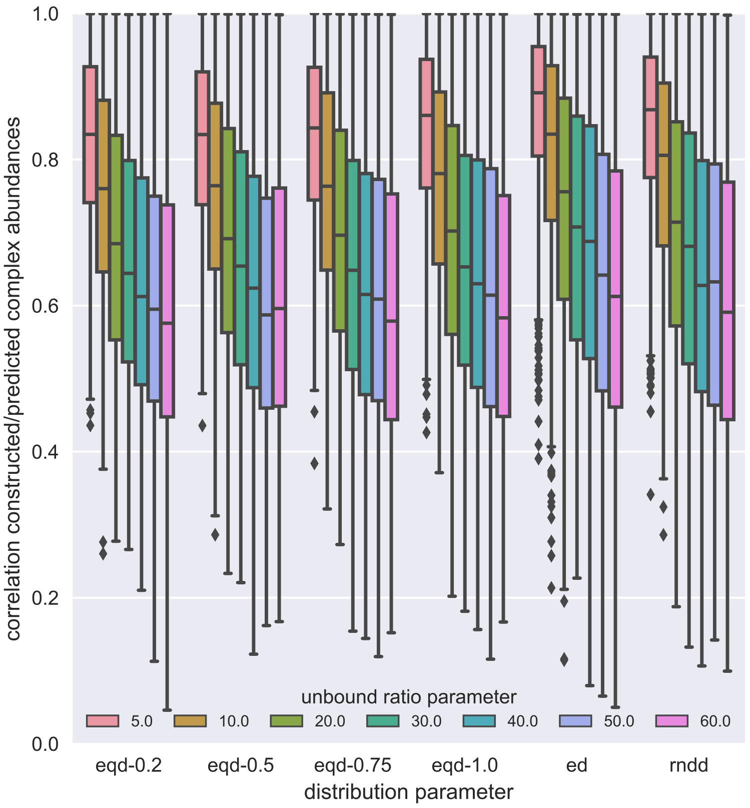

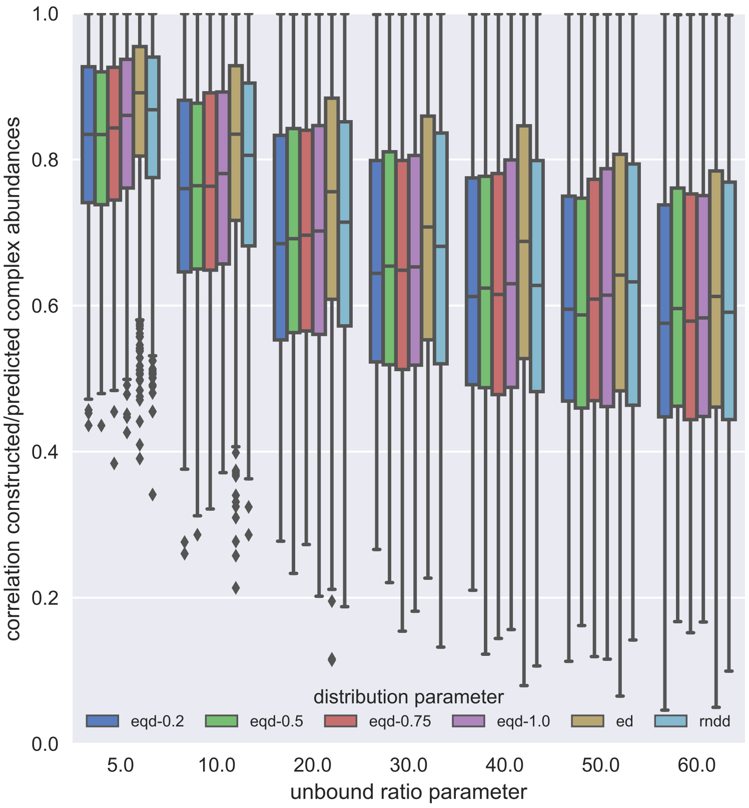

Figure S6: *Correlation of constructed complex abundances in the hypothetical exact state and CompleXChange approximations for a wider range of modeling parameters. Results are shown in dependency of the distribution parameter (left) and the unbound ratio parameter (right).*

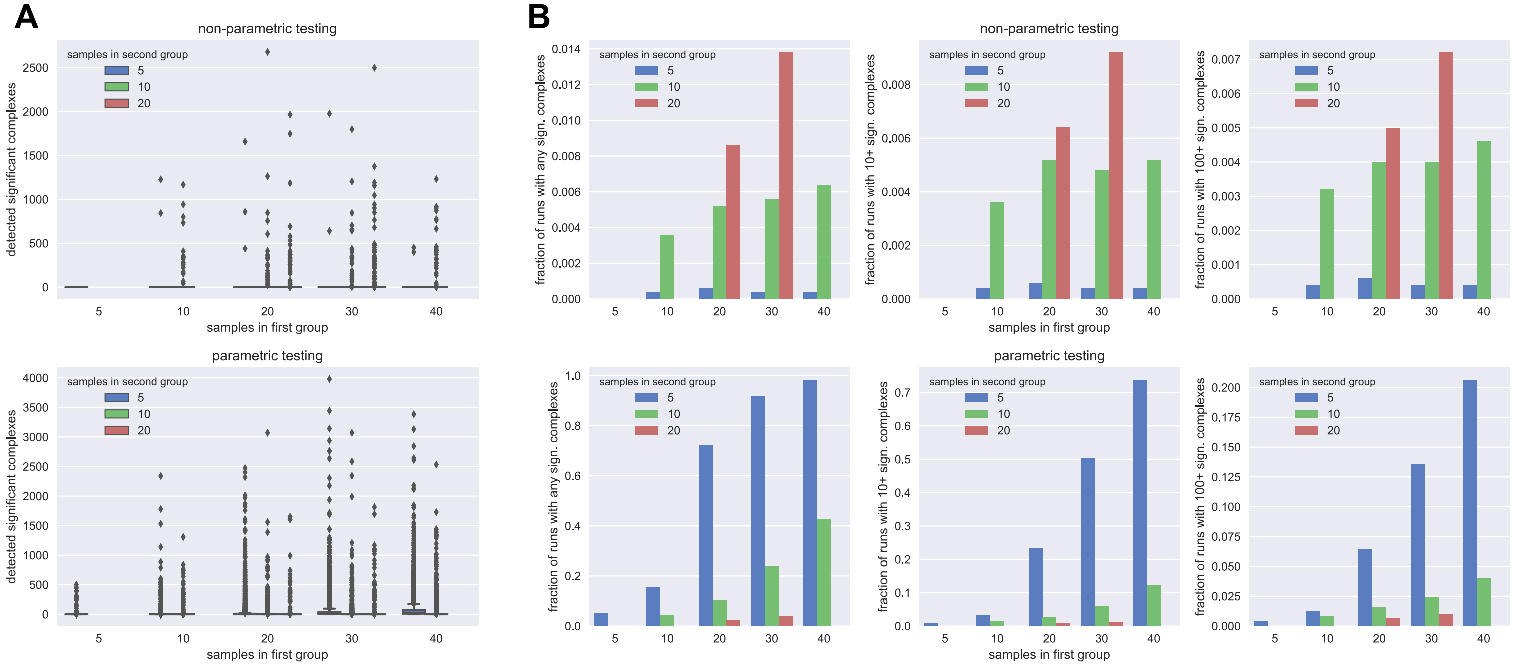

Figure S7: *Random sampling analysis on homogeneous GEUVADIS data. Here, we compare two groups of samples and how robust CompleXChange was regarding false positive complexes depending on the size of the two groups. The size of the first group is shown on the x-axis (5-40). The size of the second group varies between 5, 10 and 20 (ordered from left to right on the corresponding x-axis column). All combinations of differently sized subsets (40/20 was not tested because the data only consisted of 58 samples) were tested with CompleXChange using non-parametric (upper plots) and parametric unpaired statistical testing (lower plots). Shown are distributions of the numbers of detected complexes per random sample (A) and the fractions of tested samples with at least any/10/100 reported deregulated complexes (B).*

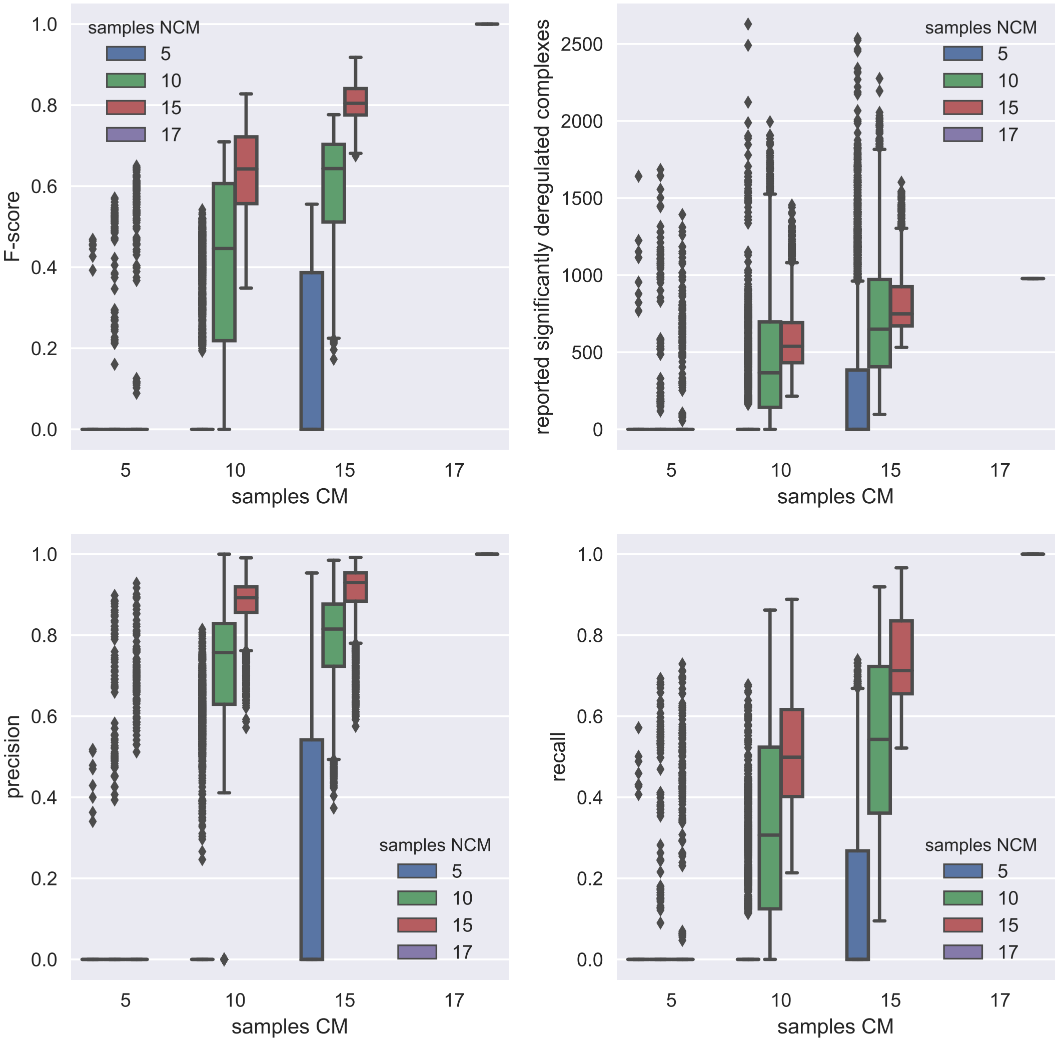

Figure S8: *Performance metrics for subsampling analysis of CompleXChange application to classical (CM) and non-classical monocytes (NCM) with unpaired data and non-parametric testing. Shown on the x-axis is the number of CM samples. The number of NCM samples is marked in the corresponding colors. Values are reported relative to the results for the full set of 17 CM and 17 NCM samples. Quality metrics of these results were thus set to 1.0.*

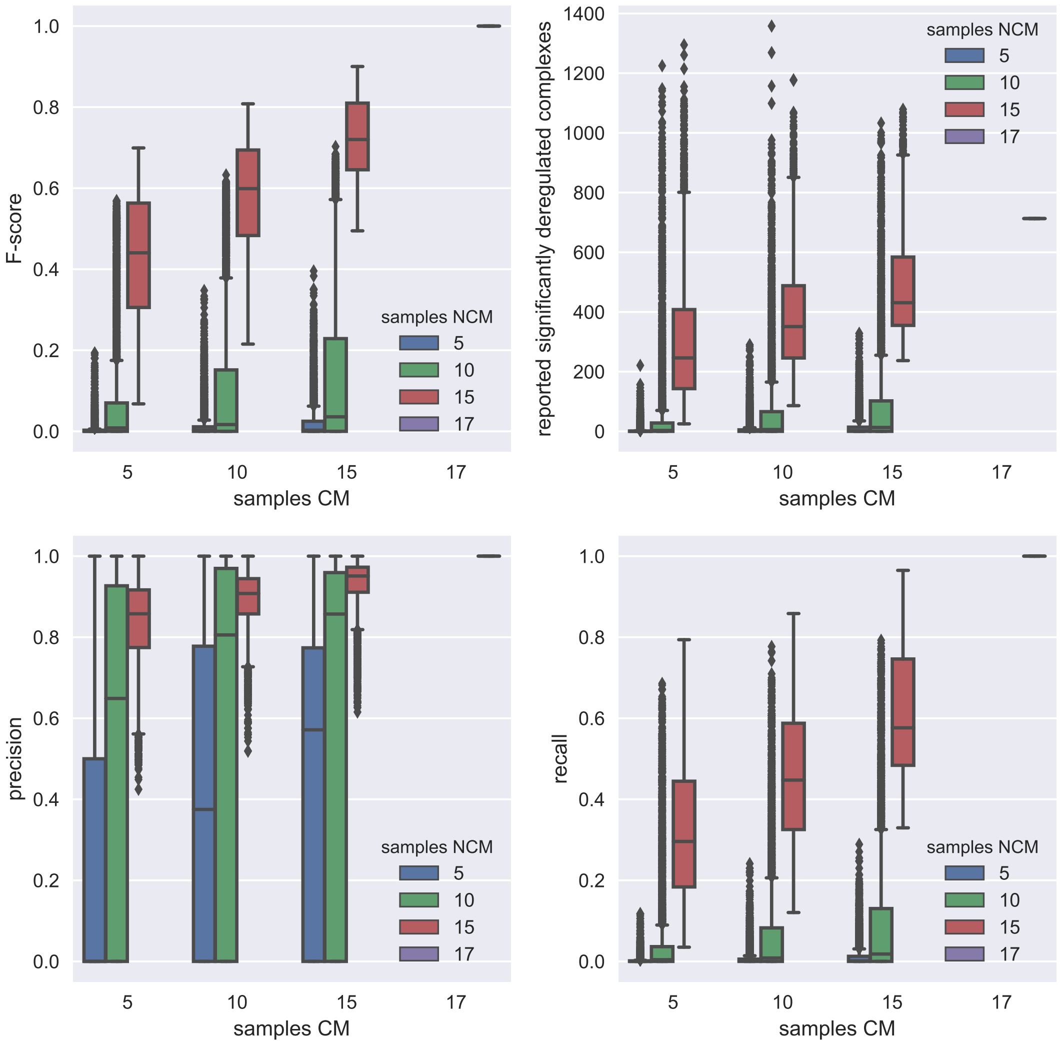

Figure S9: *Performance metrics for subsampling analysis of CompleXChange application to classical (CM) and non-classical monocytes (NCM) with unpaired data and parametric testing. Shown on the x-axis is the number of CM samples. The number of NCM samples is marked in the corresponding colors. Values are reported relative to the results for the full set of 17 CM and 17 NCM samples. Quality metrics of these results were thus set to 1.0.*

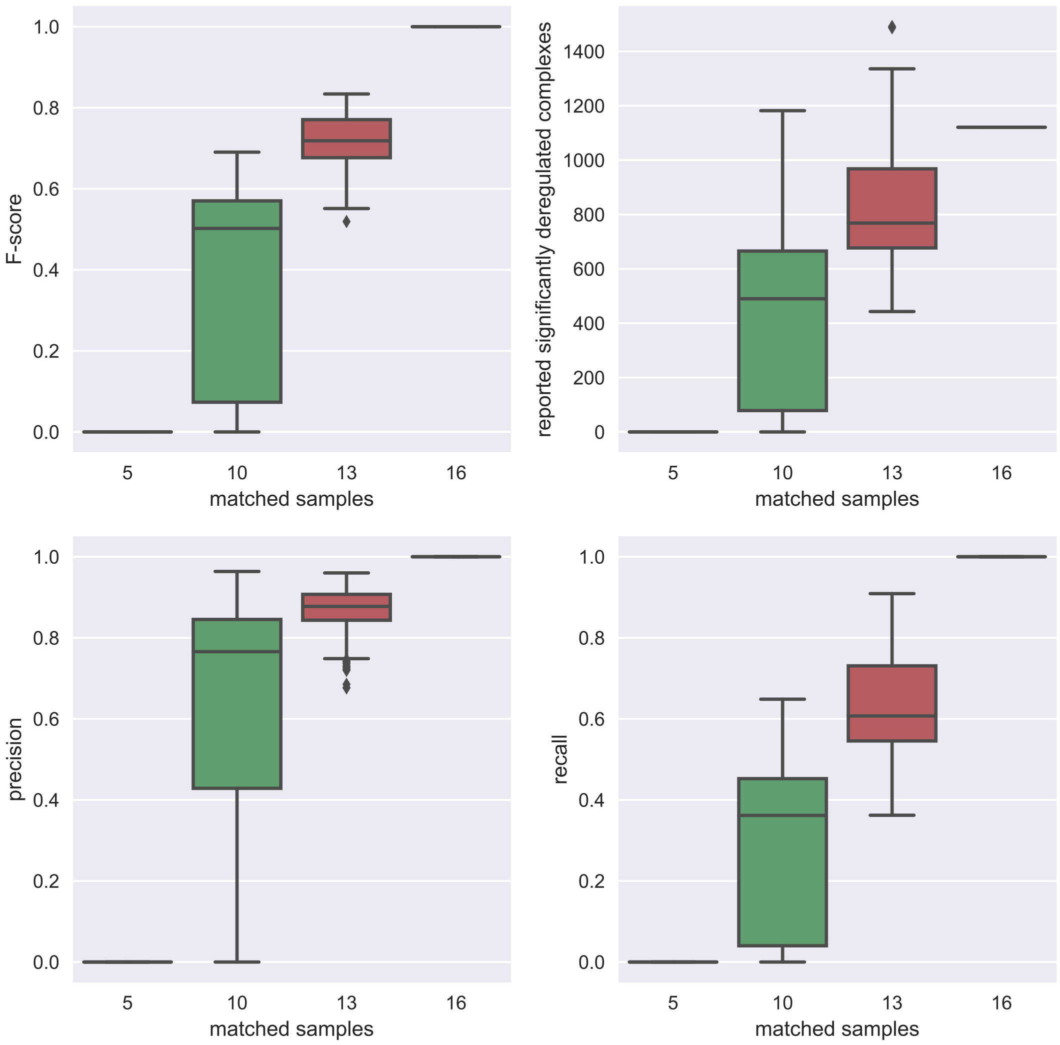

Figure S10: *Performance metrics for subsampling analysis with CompleXChange applied to data on classical and non-classical monocytes matched by donor and non-parametric testing. Values are reported relative to the results for the full set of 16 matched samples. Quality metrics of these results were thus set to 1.0.*

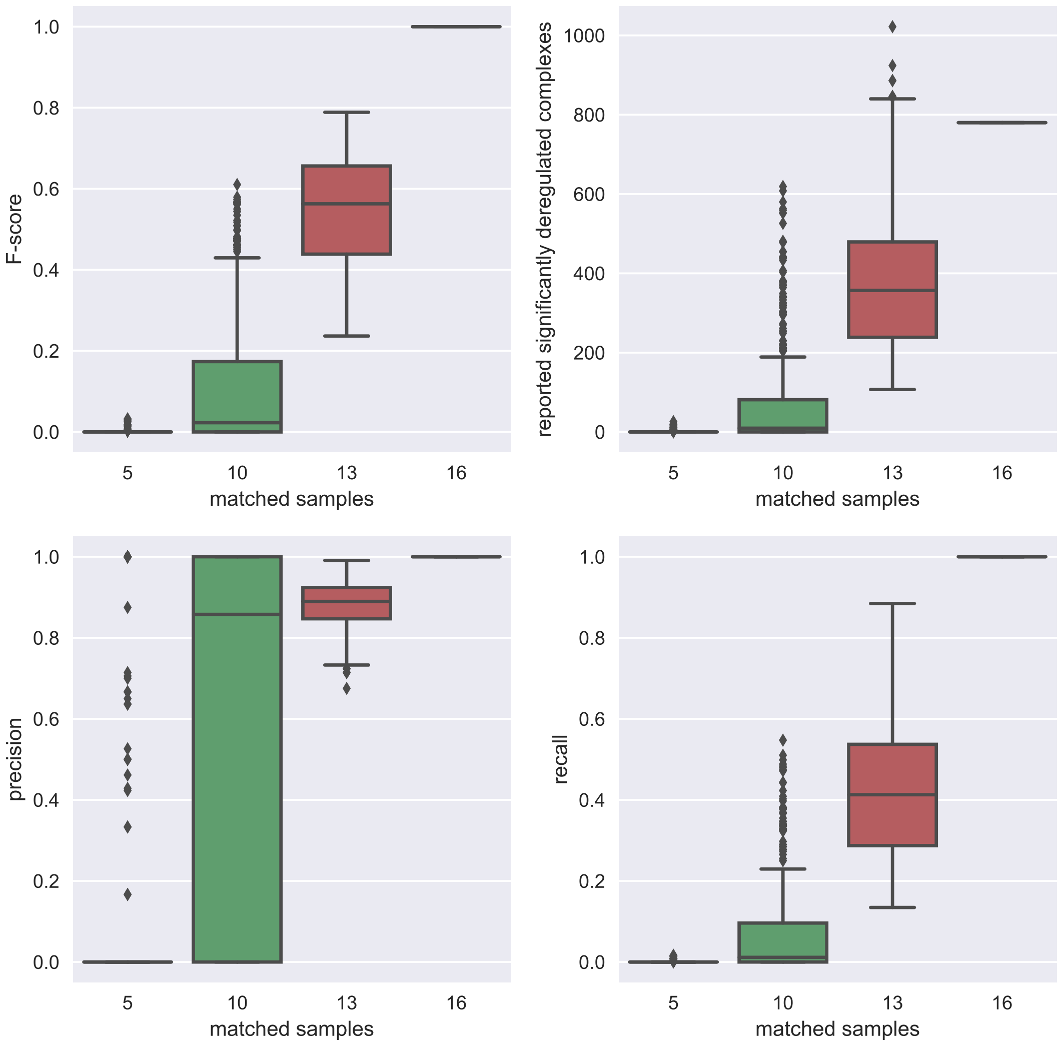

Figure S11: *Performance metrics for subsampling analysis with CompleXChange applied to data on classical and non-classical monocytes matched by donor and parametric testing. Values are reported relative to the results for the full set of 16 matched samples. Quality metrics of these results were thus set to 1.0.*

S4 Supplementary Tables

parameter set	$\Delta_{\text{median correlation}}$	p-value
eqd-0.2/5.0	0.04	$4.2 * 10^{-17}$
eqd-0.2/30.0	0.12	$1.5 * 10^{-59}$
eqd-0.2/60.0	0.13	$8.9 * 10^{-59}$
eqd-1.0/5.0	0.06	$6.3 * 10^{-33}$
eqd-1.0/30.0	0.13	$2.2 * 10^{-54}$
eqd-1.0/60.0	0.14	$1.0 * 10^{-59}$
ed/5.0	0.03	$9.4 * 10^{-15}$
ed/30.0	0.12	$4.1 * 10^{-42}$
ed/60.0	0.14	$5.0 * 10^{-51}$
rndd/5.0	0.04	$4.0 * 10^{-20}$
rndd/30.0	0.13	$8.5 * 10^{-49}$
rndd/60.0	0.14	$4.0 * 10^{-53}$

Table S1: *Median correlation differences and p-values of two-tailed Wilcoxon rank-sum tests comparing the distributions of correlation results for the CompleXChange approximation and the LP approach per construction parameter set.*

rank	TF	direction	q-value	DE rank (q-value)	DE rank (fold-change)
1.	NR4A1	+	$1.8 * 10^{-05}$	8.	22.
2.	NR1H2	+	$2.3 * 10^{-05}$	5.	9.
3.	RELA	+	$3.5 * 10^{-05}$	n.s.	n.s.
4.	ATF3	-	$6.7 * 10^{-05}$	n.s.	n.s.
5.	NR1D1	+	$7 * 10^{-05}$	2.	1.
6.	RUNX1	-	$8.4 * 10^{-05}$	27.	63.
7.	RUNX2	-	0.00011	32.	18.
8.	RXRB	+	0.00013	31.	15.
9.	JUND	-	0.00017	14.	45.
10.	EGR1	-	0.00017	3.	2.
11.	CDC5L	+	0.00022	n.s.	n.s.
12.	JUN	-	0.00034	12.	13.
13.	HMGA1	-	0.00034	n.s.	n.s.
14.	PRDM1	-	0.001	13.	3.
15.	RUNX3	-	0.0011	n.s.	n.s.
16.	GFI1B	-	0.0014	48.	64.
17.	JUNB	-	0.0025	n.s.	n.s.
18.	NFKB2	+	0.003	72.	58.
19.	THRA	+	0.0031	n.s.	n.s.
20.	MYC	-	0.0054	n.s.	n.s.
21.	CEBPB	-	0.0069	n.s.	n.s.
22.	CTCF	+	0.0074	29.	11.
23.	STAT6	+	0.011	11.	34.
24.	SMAD3	-	0.02	n.s.	n.s.
25.	NFYA	-	0.021	n.s.	n.s.
26.	KLF4	-	0.026	35.	62.
27.	NFKB1	-	0.029	n.s.	n.s.
28.	STAT3	+	0.03	43.	67.
29.	SMAD1	+	0.032	67.	55.
30.	MECP2	+	0.033	37.	27.
31.	REL	+	0.034	60.	77.
32.	NR2C2	+	0.036	n.s.	n.s.
33.	FOS	-	0.046	22.	68.
34.	MAFF	-	0.047	6.	30.
35.	STAT2	+	0.049	n.s.	n.s.

Table S2: *Transcription factors enriched in up- or down-regulated complexes. DE rank denotes the rank of the TF among all DE TFs sorted by either likelihood ratio test q-value or absolute log. fold-change as reported by sleuth. "n.s." abbreviates: not significant in differential expression analysis.*

pathway term	q-value	fold-enrichment
CCKR signaling map	0.049	3.54

***Table S3:** Enrichment analysis results for differentially expressed TFs using PANTHER and PANTHER pathway annotations.*

pathway term	q-value	fold-enrichment	associated genes
Cadmium induces DNA synthesis and proliferation in macrophages	0.0005	17.14	FOS,JUN,MYC,NFKB1,RELA
Role of EGF Receptor Transactivation by GPCRs in Cardiac Hypertrophy	0.0005	17.14	FOS,JUN,MYC,NFKB1,RELA

***Table S4:** Enrichment analysis results for CompleXChange enriched TFs using GeneTrail2 and BioCarta annotations.*

pathway term	q-value	fold-enrichment	associated genes
Chagas disease (American trypanosomiasis)	0.0026	14.29	FOS,JUN,NFKB1,RELA,SMAD3
MAPK signaling pathway	0.0026	6.86	FOS,JUN,JUND,MYC,NFKB1,NFKB2,NR4A1,RELA
Osteoclast differentiation	0.0026	6.86	FOS,JUN,JUNB,JUND,NFKB1,NFKB2,RELA,STAT2
Salmonella infection	0.0043	17.14	FOS,JUN,NFKB1,RELA
TNF signaling pathway	0.0064	8.57	CEBPB,FOS,JUN,JUNB,NFKB1,RELA
Epstein-Barr virus infection	0.0095	7.91	JUN,MYC,NFKB1,NFKB2,RELA,STAT3
Hepatitis B	0.0097	4.68	FOS,JUN,MYC,NFKB1,RELA,SMAD3,STAT2,STAT3,STAT6
Inflammatory bowel disease (IBD)	0.023	6.43	JUN,NFKB1,RELA,SMAD3,STAT3,STAT6
Leishmaniasis	0.023	11.43	FOS,JUN,NFKB1,RELA
Pathways in cancer	0.023	3.73	FOS,JUN,MYC,NFKB1,NFKB2,RELA,RUNX1,RXRB,SMAD3,STAT3
Epithelial cell signaling in Helicobacter pylori infection	0.029	17.14	JUN,NFKB1,RELA
Adipocytokine signaling pathway	0.033	9.80	NFKB1,RELA,RXRB,STAT3
B cell receptor signaling pathway	0.033	9.80	FOS,JUN,NFKB1,RELA
Chemokine signaling pathway	0.033	9.80	NFKB1,RELA,STAT2,STAT3
Pertussis	0.033	9.80	FOS,JUN,NFKB1,RELA
T cell receptor signaling pathway	0.033	9.80	FOS,JUN,NFKB1,RELA
HTLV-I infection	0.038	3.59	ATF3,EGR1,FOS,JUN,MYC,NFKB1,NFKB2,RELA,SMAD3
Chronic myeloid leukemia	0.045	6.59	MYC,NFKB1,RELA,RUNX1,SMAD3

***Table S5:** Enrichment analysis results for CompleXChange enriched TFs using GeneTrail2 and KEGG annotations.*

pathway term	q-value	fold-enrichment	associated genes
Senescence-Associated Secretory Phenotype (SASP)	0.00026	17.14	CEBPB,FOS,JUN,NFKB1,RELA,STAT3

***Table S6:** Enrichment analysis results for CompleXChange enriched TFs using GeneTrail2 and Reactome annotations.*

pathway term	q-value	fold-enrichment	associated genes
Oncostatin M Signaling Pathway	0.00011	10.55	CEBPB,EGR1,FOS,JUNB,JUND,NFKB1,RELA,STAT3
TGF-beta Receptor Signaling	0.0005	8.57	FOS,JUN,NFKB1,RUNX2,RUNX3,SMAD1,SMAD3,STAT3
TNF alpha Signaling Pathway	0.0045	17.14	JUN,NFKB1,NFKB2,REL
TSLP Signaling Pathway	0.0045	9.35	MYC,NFKB1,NFKB2,RELA,STAT3,STAT6
Androgen receptor signaling pathway	0.0077	7.35	JUN,NR2C2,RELA,RUNX2,SMAD3,STAT3
IL-1 signaling pathway	0.0077	13.71	JUN,NFKB1,REL,RELA
IL-4 Signaling Pathway	0.0077	7.35	CEBPB,FOS,NFKB1,RELA,STAT3,STAT6
MAPK Signaling Pathway	0.0077	7.35	FOS,JUN,JUND,MYC,NFKB1,NR4A1
Regulation of toll-like receptor signaling pathway	0.0077	9.52	FOS,JUN,NFKB1,NFKB2,RELA
TGF-beta Signaling Pathway	0.0077	5.49	ATF3,FOS,JUN,JUNB,JUND,MYC,RUNX2,SMAD3
TSH signaling pathway	0.0077	9.52	EGR1,FOS,JUN,MYC,STAT3
TWEAK Signaling Pathway	0.0077	13.71	JUN,NFKB1,NFKB2,RELA
Toll-like Receptor Signaling Pathway	0.0077	9.52	FOS,JUN,NFKB1,NFKB2,RELA
RANKL/RANK Signaling Pathway	0.014	8.57	FOS,JUN,NFKB1,NFKB2,RELA
Corticotropin-releasing hormone	0.015	6.43	FOS,JUNB,JUND,NFKB1,NR4A1,RELA
IL17 signaling pathway	0.015	11.43	CEBPB,NFKB1,RELA,STAT3
EBV LMP1 signaling	0.017	17.14	NFKB1,NFKB2,RELA
Selenium Micronutrient Network	0.017	17.14	NFKB1,NFKB2,RELA
TCR Signaling Pathway	0.017	7.79	FOS,JUN,NFKB1,REL,RELA
Vitamin B12 Metabolism	0.017	17.14	NFKB1,NFKB2,RELA
BDNF signaling pathway	0.017	6.05	EGR1,FOS,JUN,NFKB1,RELA,STAT3
Myometrial Relaxation and Contraction Pathways	0.039	6.59	ATF3,FOS,JUN,MAFF,NFKB1
IL-2 Signaling Pathway	0.044	8.57	FOS,JUN,MYC,STAT3
Rac1/Pak1/p38/MMP-2 pathway	0.044	8.57	MYC,NFKB1,RELA,STAT3
AGE/RAGE pathway	0.046	6.12	JUN,NFKB1,RELA,SMAD3,STAT3
Apoptosis Modulation and Signaling	0.046	12.86	FOS,JUN,NFKB1
Folate Metabolism	0.046	12.86	NFKB1,NFKB2,RELA
Signaling of Hepatocyte Growth Factor Receptor	0.046	12.86	FOS,JUN,STAT3

Table S7: *Enrichment analysis results for CompleXChange enriched TFs using GeneTrail2 and WikiPathways annotations.*

pathway term	q-value	fold-enrichment
Inflammation mediated by chemokine and cytokine signaling pathway	$7.5 * 10^{-05}$	9.95
Gonadotropin-releasing hormone receptor pathway	0.00052	4.42
Apoptosis signaling pathway	0.0016	7.28
CCKR signaling map	0.0017	5.66
Toll receptor signaling pathway	0.0028	11.05
TGF-beta signaling pathway	0.0052	8.84
B cell activation	0.015	8.84
T cell activation	0.017	8.84
Interleukin signaling pathway	0.024	5.20
PDGF signaling pathway	0.034	3.79

Table S8: *Enrichment analysis results for CompleXChange enriched TFs using PANTHER and PANTHER pathway annotations.*

significance level	p-value (all complexes)	p-value (filtered complexes)
0.05	0.0001	0.0001
0.01	0.0001	0.0001
0.001	0.0003	0.0002

Table S9: *Results of permutation tests to assess the significance of CV accuracies of CompleXChange results of varying stringency with random complex sets of equivalent size. Since* 10^4 *iterations were made,* $p = 10^{-4}$ *represents the lower limit.*

References

[1] A. Mortazavi et al. Mapping and quantifying mammalian transcriptomes by RNA-Seq. *Nat. Methods*, 5(7):621–628, Jul 2008.

[2] S. Zhao et al. Comparison of RNA-Seq and microarray in transcriptome profiling of activated T cells. *PLoS ONE*, 9(1):e78644, 2014.

[3] P. L. Kastritis and A. M. Bonvin. On the binding affinity of macromolecular interactions: daring to ask why proteins interact. *J R Soc Interface*, 10(79):20120835, Feb 2013.

[4] H. Pimentel et al. Differential analysis of RNA-seq incorporating quantification uncertainty. *Nat. Methods*, 14(7):687–690, Jul 2017.

[5] D. Stockel et al. Multi-omics enrichment analysis using the GeneTrail2 web service. *Bioinformatics*, 32(10):1502–1508, 05 2016.

[6] H. Mi et al. PANTHER version 11: expanded annotation data from Gene Ontology and Reactome pathways, and data analysis tool enhancements. *Nucleic Acids Res.*, 45(D1):D183–D189, Jan 2017.
